# Investigating the Cytoprotective Mechanisms of the Tardigrade Damage Suppressor (Dsup) Protein in Human Cells Under Hypoxic Stress

**DOI:** 10.3390/ijms262110452

**Published:** 2025-10-28

**Authors:** Enxhi Shaba, Claudia Ricci, Lorenza Vantaggiato, Maria Francesca Paolocci, Tommaso Regoli, Kateryna Miedviedieva, Jlenia Brunetti, Valerio Ciccone, Claudia Cecchin, Sandra Donnini, Carlotta Marzocchi, Claudia Landi, Silvia Cantara

**Affiliations:** 1 Functional Proteomics Lab, Department of Life Sciences, University of Siena, 53100 Siena, Italy; enxhi.shaba@unisi.it (E.S.); lorenz.vantaggiato2@unisi.it (L.V.); claudia.landi@unisi.it (C.L.); 2 Department of Medical Surgical and Neurological Sciences, University of Siena, 53100 Siena, Italy; claudia.ricci@unisi.it (C.R.); tommaso.regoli@gmail.com (T.R.); kate018535@gmail.com (K.M.); 3 Department of Medical Biotechnologies, University of Siena, 53100 Siena, Italy; mariafrancesca.pa@student.unisi.it (M.F.P.); jlenia.brunetti@unisi.it (J.B.); 4 Pharmacology Lab, Department of Life Sciences, University of Siena, 53100 Siena, Italy; valerio.ciccone@unisi.it (V.C.); c.cecchin@student.unisi.it (C.C.); sandra.donnini@unisi.it (S.D.); 5 UOC Laboratorio Patologia Clinica, AOU Senese, 53100 Siena, Italy; carlotta.marzocchi@ao-siena.toscana.it

**Keywords:** Dsup, ischemia, reperfusion, oxidative stress, autophagy, proteomics

## Abstract

Ischemia/reperfusion injury (IRI) is a common damage due to the restoration of blood flow following an ischemic injury. Its pathogenesis is mainly linked to the production of reactive oxygen species (ROS), which sustain cell damage and promote cell death. The tardigrade damage suppressor protein (Dsup) is a DNA-binding protein that enables tardigrades to tolerate stress conditions, including oxidative stress. We investigated the ability of the Dsup to protect human cells from IRI, using an in vitro model of hypoxia and reoxygenation. We exposed HEK293TT cells transfected with the Dsup to hypoxic injury and analyzed cell viability, oxidative stress, expression of antioxidant proteins using functional assays, and a proteomic approach to dissect the molecular mechanisms modulated by the Dsup. Dsup expression significantly enhanced cell survival following hypoxia-reoxygenation and markedly reduced intracellular ROS levels. Proteomic and Western blot analyses revealed a significant upregulation of antioxidant enzymes in Dsup-expressing cells. Furthermore, the Dsup modulated autophagy and key stress-related pathways, including the MAPK cascade. This study demonstrates that the Dsup protects human cells from IRI by reducing oxidative stress and modulating key cytoprotective pathways. Our results establish the Dsup as a promising candidate for future therapeutic applications against IRI, meriting further exploration in in vivo models.

## 1. Introduction

Ischemia refers to the restriction of the blood supply to tissues, resulting in a deficiency of oxygen and nutrients needed for cellular metabolism. During ischemia, the lack of oxygen (hypoxia) and nutrients terminates cellular respiration, forcing cells to switch to anaerobic metabolism. This leads to the accumulation of lactic acid, which lowers the pH within cells and causes metabolic acidosis [1]. The lack of ATP impairs the function of ion pumps, contributing to cellular swelling and membrane damage [2]. While the primary aim during an ischemic event is to restore blood flow, the process of reperfusion can paradoxically exacerbate tissue damage. This phenomenon is known as ischemia–reperfusion injury (IRI), and it is mainly sustained by the production of reactive oxygen species (ROS) at the ischemic site [3]. Upon reperfusion, the flood of oxygen activates xanthine oxidase, which degrades hypoxanthine, releasing the reactive superoxide anion, which is then converted into hydrogen peroxide (H_2_O_2_) and hydroxyl radical [4]. The primary effects of this process are cell membrane lipid peroxidation, the production and release of proinflammatory molecules, the disruption of cell permeability, and ultimately cell death. Ischemia can occur in different organs, such as the brain, myocardium, and kidneys, and it represents a common problem in transplants.

Several therapeutic strategies have been explored to reduce the impact of IRI. First, the use of antioxidant vitamins has been proposed [5,6,7,8,9,10]. Another approach is represented by ischemic preconditioning (IPC), which involves exposing tissues to brief periods of ischemia followed by reperfusion before a prolonged ischemic event [11,12,13]. Hypothermia can reduce the generation of ROS, thereby limiting the extent of reperfusion injury, but it can cause complications such as infection and coagulation disorders, while a topical approach requires precise control and early application to be effective [14,15]. Among pharmacological agents, calcium channel blockers, anti-inflammatory agents, and inhibitors of mitochondrial permeability transition pore (MPTP) are subject to investigation [16,17,18].

The tardigrade damage suppressor protein (Dsup) is a unique protein that binds to nucleosomes, forming a barrier around chromatin and shielding it from hydroxyl radicals, which can otherwise cause significant DNA damage [19]. Recent studies have explored the broader impact of the Dsup on human cells and revealed that it activates several stress response mechanisms [20,21]. These include the DNA damage response, mRNA processing, and telomere maintenance, which contribute to the enhanced stress resistance observed in Dsup-transfected cells [21].

It is evident that the Dsup from tardigrades is an intriguing area of study with promising implications for enhancing cellular resilience in various applications, including ischemia–reperfusion injury. In this article, we exposed Dsup-transfected cells to ischemia and evaluated which pathways are activated to induce resistance not only to ischemia itself but, most importantly, to the reperfusion injury.

## 2. Results

### 2.1. Dsup Protects HEK293TT Cells Exposed to Hypoxia

**Figure 1:**
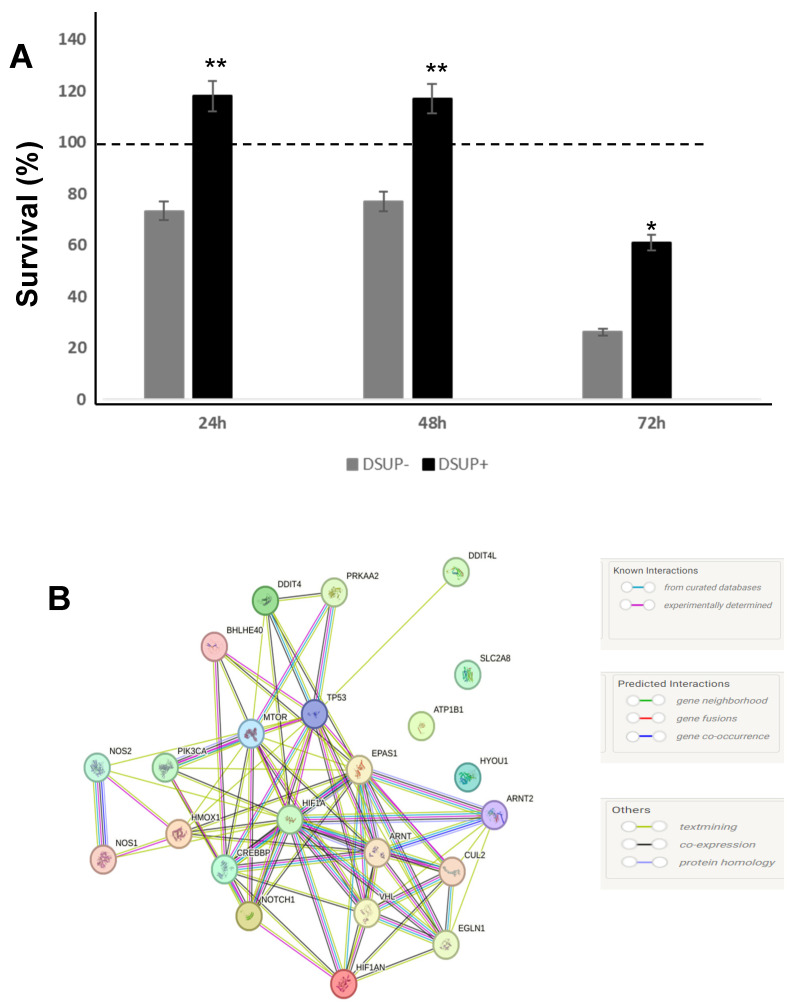

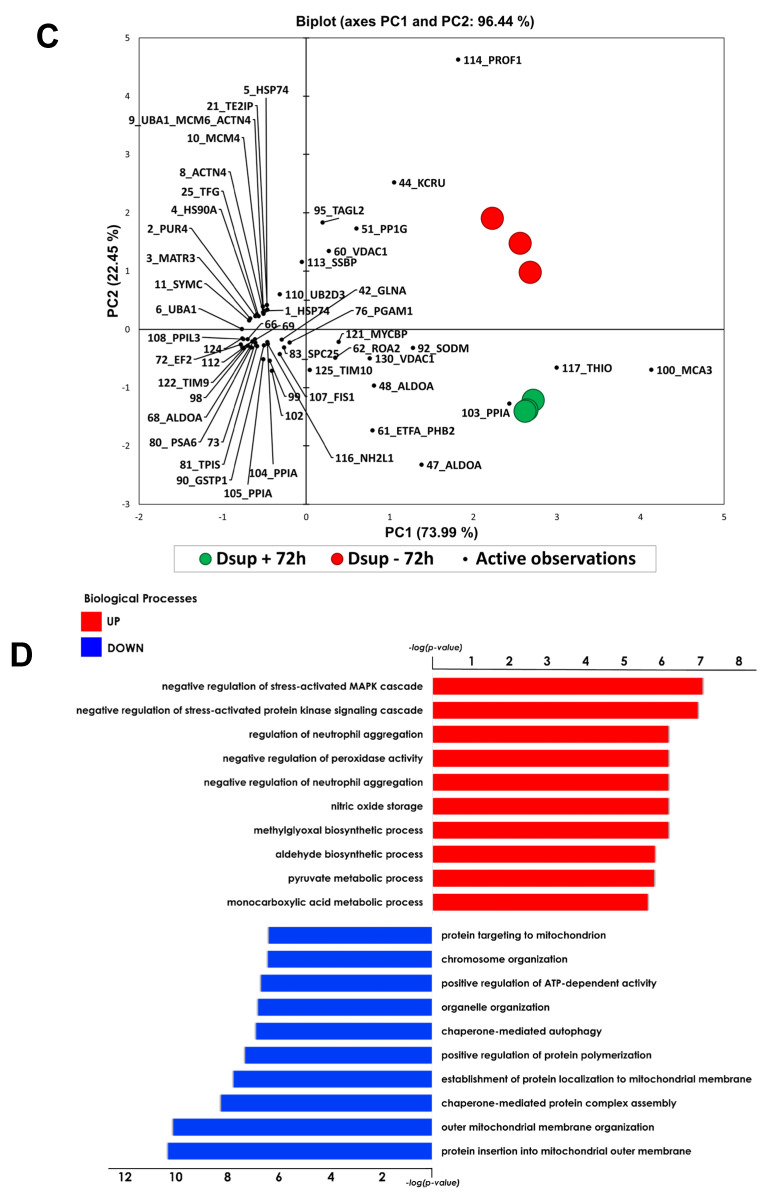
(**A**) Cell survival in Dsup− or Dsup+ cells after CoCl_2_ treatment for 24, 48, or 72 h. Results are reported as a percentage of survival compared to untreated cells (100% dotted line); (**B**) STRING protein–protein interaction network for differentially expressed genes after CoCl_2_ treatment. Both known (purple and light blue lines) and predicted interactions (blue, green, and red lines are shown); (**C**) Principal component analysis of the statistically relevant spots obtained by the differential proteomic analysis between Dsup− and Dsup+ after 72 h of hypoxic stress. PCA includes 96.44% of the variance. Differential spots are marked in black, while samples are colored in red for Dsup− and in green for Dsup+. (**D**) Enrichment analysis by GO biological processes by MetaCore software v. 6.37 of differential spots between Dsup− and Dsup+ after 72 h of hypoxic stress. Proteomic data are considered as two datasets: red bars indicate the significance (*p*-value in −log_10_) of the most enriched biological processes by up-regulated proteins in Dsup+, while blue bars indicate the down-regulated proteins in Dsup+. * *p* < 0.05; ** *p* < 0.01.

To test whether the Dsup transfection confers protection against hypoxia, we exposed Dsup+ and Dsup− cells to 100 μM cobalt chloride (CoCl_2_) (22) for 24, 48, and 72 h. As shown in Figure 1A, Dsup+ cells exposed to hypoxia showed an increased survival rate at each time point. After 72 h of hypoxia, cells tended to die, but death extension was lower when compared to Dsup− cells (*p* < 0.01). The mechanisms of this protection may be linked to the increased transcription of genes involved in hypoxia-responsive mechanisms. In fact, when analyzed by real-time PCR after 72 h of hypoxia, in Dsup+ cells, we observed an increased fold change of approximately 1 of more than 20 hypoxia response-related genes belonging to these classes: transcription factors, immune and stress response, signal transduction, apoptosis, oxidoreductases, proteases, and protein metabolism as well as induction of HIF1α (Table 1). Networking among these proteins evaluated with STRING is shown in Figure 1B.

We also performed a proteomic analysis on these cells, and an average of 3200 spots per gel was detected by image analysis software. Only spots with a *p*-value ≤ 0.05 and a fold change ≥ 2 were considered. This differential analysis highlighted 54 differentially abundant spots, of which 19 were down and 35 were upregulated (Figure S1 and Table S1). Of 54 spots, 45 proteins were identified by MALDI ToF mass spectrometry. We performed a principal component analysis (PCA) of the %V data of the differential spots to easily identify the largest variation in the data. This revealed a differential protein pattern between Dsup+ and Dsup− cells after 72 h of hypoxia, since samples have a different distribution into the 2D plane (Figure 1C, green and red dots, respectively). PROF1, KVRU, MCA3, THIO, PPIA, ALDOA, EFTA, and NH2L1 protein abundance seem to have the strongest role in this sample separation. Enrichment analysis by MetaCore software, by Gene Ontology (GO) Biological Processes (BPs), reveals that up-regulated proteins in Dsup+ after 72 h of CoCl_2_ are involved in the negative regulation of stress-activated MAPK cascade, negative regulation of neutrophil aggregation, negative regulation of peroxidase activity, nitric oxide storage, methylglyoxal biosyntetic process, aldheyde biosyntetic process, piruvate metabolic process, and mono carboxylic acid metabolic process, while down-regulated proteins act on protein insertion and organization into outer mitochondrial membrane, chaperone-mediated protein complex assembly, positive regulation of protein polymerization, chaperone-mediated autophagy, organelle organization, positive regulation of ATP-dependent activity and chromosome organization (Figure 1D).

### 2.2. Dsup-Induced Mechanisms to Protect Human Cells from Ischemia–Reperfusion Injury

**Figure 2:**
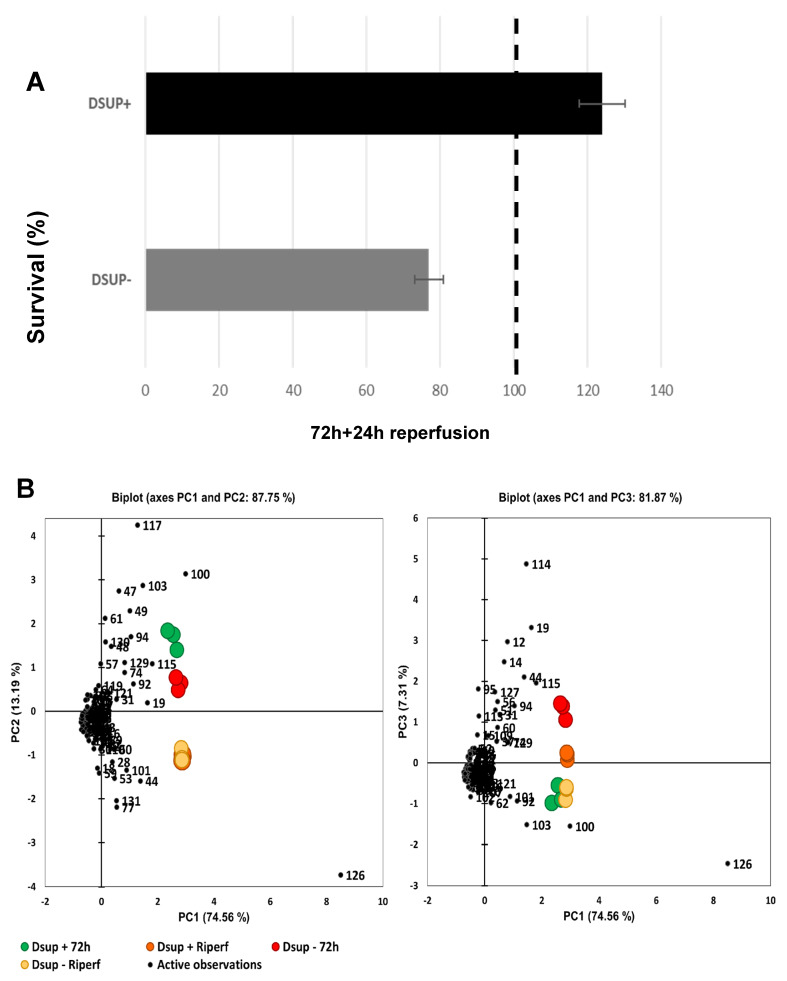

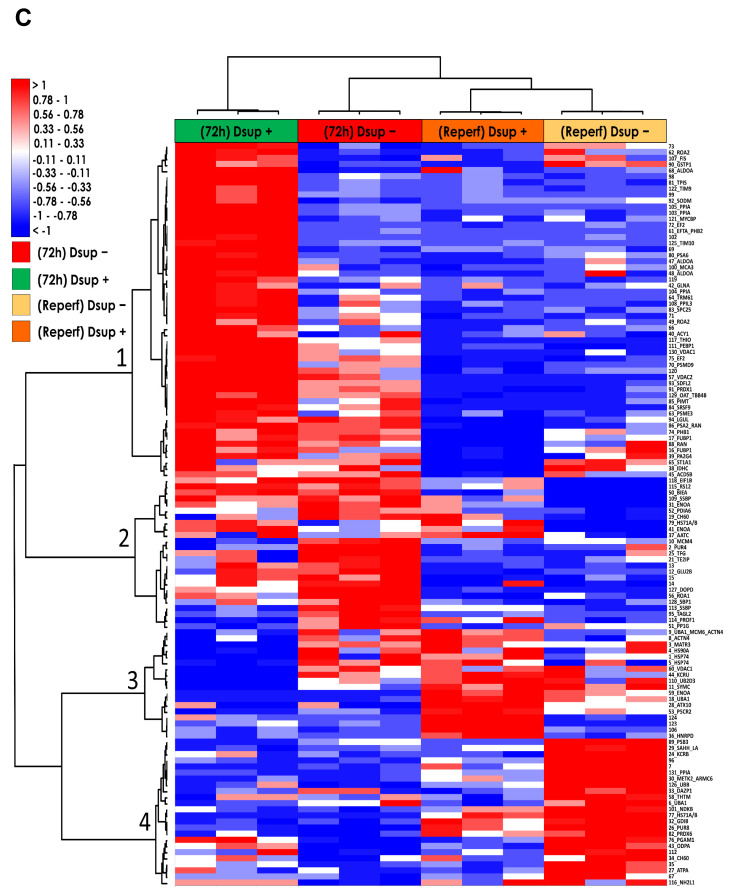

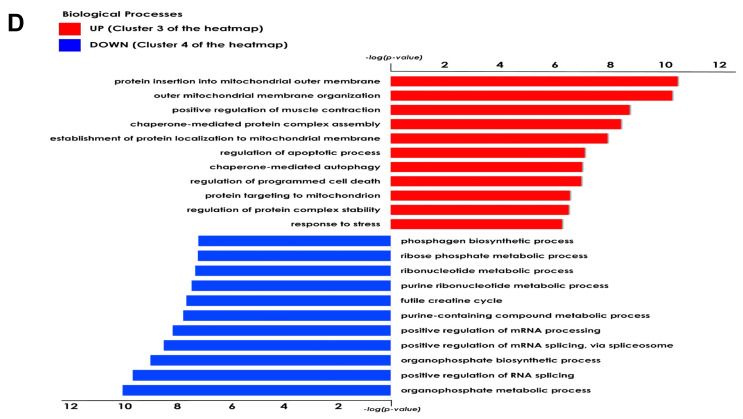
(**A**) MTT assay after ischemia–reperfusion injury in Dsup+ and Dsup− cells after reperfusion. Results are reported as percentage of survival compared to untreated cells (100% dotted line); (**B**) Principal component analysis of the statistically relevant spots obtained by the differential proteomic analysis considering Dsup− and Dsup+ after 72 h of hypoxic stress, as well as Dsup− and Dsup+ after 24 h of recovery in normal O_2_ conditions (reperfusion). On the left, PCA shows the distribution of samples along PC1 and PC2, summarizing 87.75% of variance, while on the right, the graph shows the distribution of samples along PC1 and PC3, summarizing 81.87% of variance. Differential spots are marked in black, while samples are colored in red for (72 h) Dsup−, in green for (72 h) Dsup+, in yellow for (Reperf) Dsup−, and in orange for (Reperf) Dsup+. (**C**) Heatmap analysis of Dsup− and Dsup+ after 72 h of hypoxic stress, as well as Dsup− and Dsup+ after 24 h of recovery in normal O_2_ conditions (reperfusion). The same colors distinguishing different conditions are used as in PCA. As shown in the legend, up-regulated spots are reported in red, while down-regulated spots are reported in blue. Analysis results in four main clusters, indicated in the dendrogram on the left. (**D**) Enrichment analysis by GO biological processes by MetaCore software v. 6.37 of up-regulated proteins (cluster three) and down-regulated proteins (cluster four) in Dsup+ after 24 h of reperfusion, as indicated in the heatmap. Red bars indicate the significance (*p*-value in −log_10_) of the most enriched biological processes by up-regulated proteins in (Reperf) Dsup+, while blue bars indicate the down-regulated proteins in (Reperf) Dsup+.

After an ischemic injury, tissue death can be linked to sudden reperfusion of the ischemic area. We simulated this event by replacing the medium containing 100 μM CoCl_2_ with complete medium (10% FCS) after 72 h of hypoxia and allowing cells to grow for 24 h (5% CO_2_, 37 °C). We then performed an MTT assay, and we observed a significantly increased survival rate in Dsup+ cells compared to Dsup− cells (Figure 2A). In Dsup−, during reperfusion, cell proliferation is balanced by cell death, and cell number returned to that measured after 48 h of hypoxia. On the contrary, in Dsup+ cells, we observed a huge increase in cell proliferation, indicating an acquired capacity of the cells to overcome reperfusion injury.

To understand the modifications at the protein level induced by reperfusion, we performed a proteomic analysis, also comparing Dsup+ and Dsup− cells after the reperfusion event. We found 38 differential spots, 25 down- and 13 up-regulated in Dsup+ cells, of which 31 were identified by mass spectrometry (Figure S1 and Table S1b). Complete proteomic data from Dsup+/− 72 h CoCl_2_ and reperfusion have been used to perform PCA and heatmap analysis to have a whole panorama of the proteins and samples’ behavior. In Figure 2B, it is reported that the PCA, PC1 vs. PC2, and PC1 vs. PC3 visualizations highlight the differential protein pattern, well distinguishing the four groups of analysis, suggesting a differential response of Dsup+ cells not only to hypoxia but also to reperfusion with respect to Dsup− cells. Moreover, heatmap analysis reported in Figure 2C shows that Dsup has an immediate impact on cell response to hypoxia, upregulating proteins in cluster one of Dsup+ cells with respect to Dsup− cells. On the other hand, during reperfusion, there is a reversal of the situation: cluster one is strongly downregulated in both Dsup+/− conditions. Characteristic protein profiles of Dsup+ cells, during reperfusion, are observed in cluster three, where proteins showed an upregulation, and cluster four, where proteins showed a downregulation with respect to Dsup− cells. Upregulated proteins in Dsup+ cells after reperfusion are involved in the insertion and organization of the mitochondria and mitochondrial membrane proteins, in chaperon-mediated protein complex assembly and autophagy, in regulation of the apoptotic process, and in response to stress (Figure 2D). Instead, the downregulated proteins in Dsup+ cells after reperfusion are involved in organophosphate metabolic processes, regulation of RNA splicing, purine-containing compound metabolic processes, the creatine cycle, ribose phosphate metabolic processes, and phosphagen biosynthetic processes (Figure 2D).

### 2.3. Dsup Protects Human Cells from ROS Injury Associated with Reperfusion

**Figure 3:**
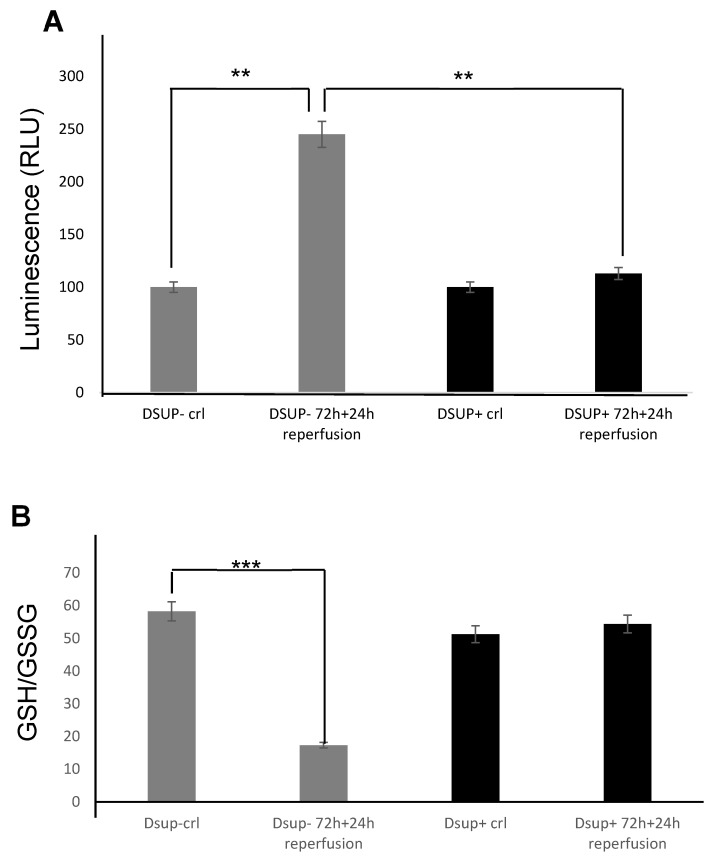
(**A**) ROS levels in Dsup− (gray boxes) and Dsup+ (black boxes) cells at basal condition and after the ischemia/reperfusion protocol. (**B**) GSH/GSSG ratio measured in Dsup− and Dsup+ cells at basal conditions and after the ischemia/reperfusion protocol. ** *p* < 0.01; *** *p* < 0.001 by one-way ANOVA test. Each experiment was run in triplicate, and each experimental point was repeated three times.

From the moment that reperfusion injury involves the generation of reactive oxygen species (ROS) that is fueled by the reintroduction of oxygen when the blood flow is reestablished, we deeply investigated molecular pathways involved in Dsup protection against H_2_O_2_ exposure (20). Firstly, we demonstrated that after 72 h of CoCl_2_ and 24 h of reperfusion, ROS production is significantly increased (*p* < 0.01) in the cell media of DSUP− cells, and the GSH/GSSG ratio is strongly decreased (*p* < 0.001), indicating a rise in oxidative stress (Supplementary Figure S2A,B). Importantly, in Dsup+ cells treated with hypoxia/reperfusion, we observed a reduction in ROS levels similar to that measured in untreated cells (Figure 3A), and the GSH/GSSG ratio was maintained at basal levels (Figure 3B).

**Figure 4:**
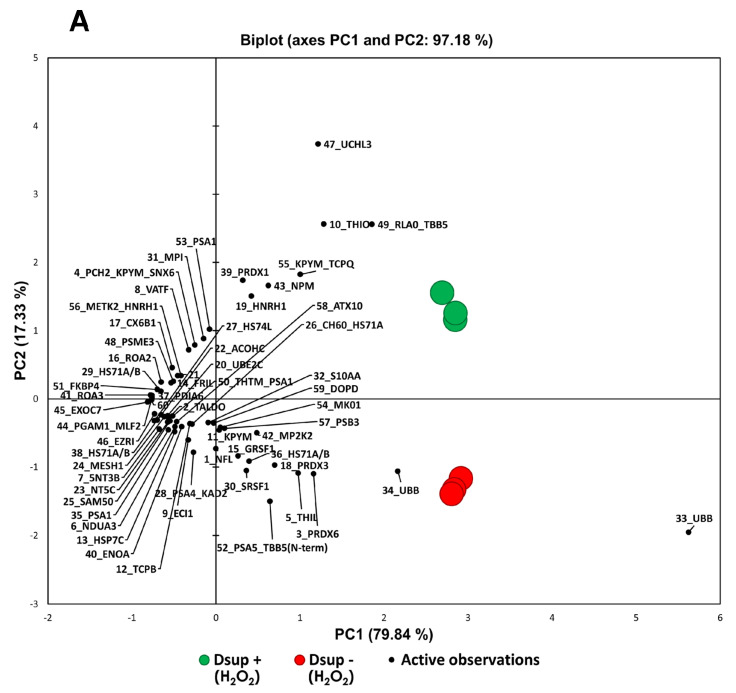

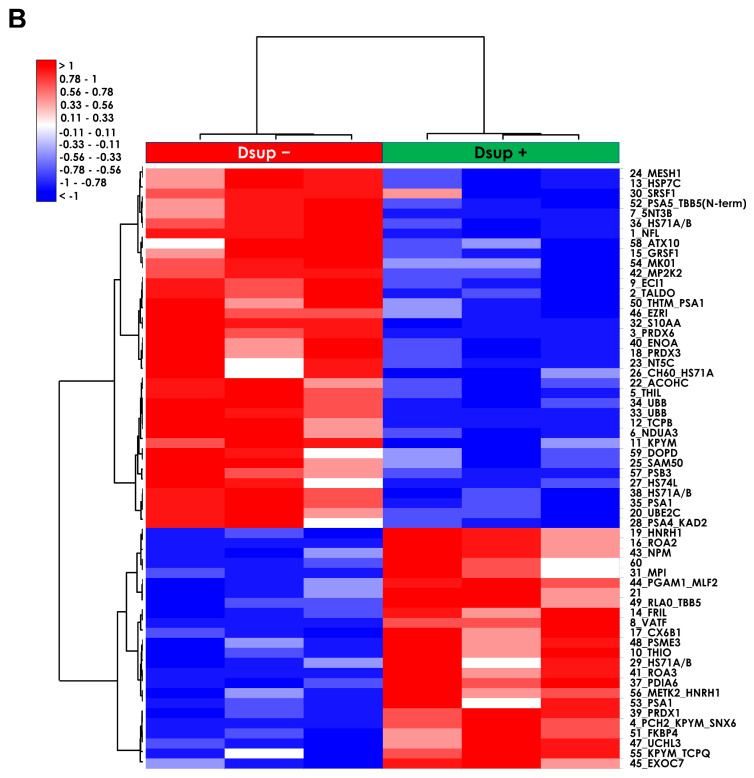

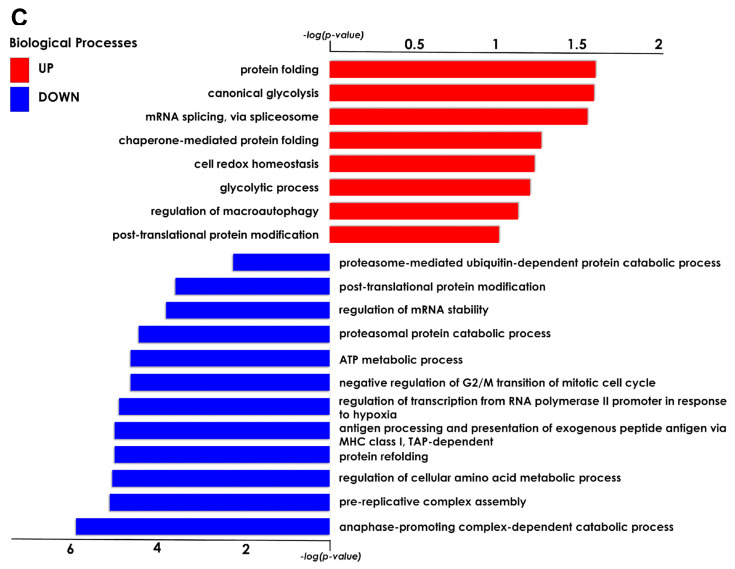
(**A**) Principal component analysis of the statistically relevant spots obtained by the differential proteomic analysis between Dsup− and Dsup+ under oxidative stress. PCA includes 97.18% of the variance. Differential spots are marked in black, while samples are colored in red for Dsup− and in green for Dsup+. (**B**) Heatmap analysis of Dsup− and Dsup+ under oxidative stress. As shown in the legend, up-regulated spots are reported in red, while down-regulated spots are reported in blue; samples are indicated in red for Dsup− and in green for Dsup+. (**C**) Enrichment analysis by GO biological processes by MetaCore software v. 6.37 of differential spots between Dsup− and Dsup+ under oxidative stress. Proteomic data are considered as two datasets: red bars indicate the significance (*p*-value in −log10) of the most enriched biological processes by up-regulated proteins in Dsup+, while blue bars indicate those for down-regulated proteins in Dsup+.

As Dsup transfection triggers an increased overall survival of human cells in response to oxidative stress (20), we applied a differential proteomic approach in Dsup− and Dsup+ cells after H_2_O_2_ treatment to investigate the molecular mechanisms by which Dsup responds to this stress and can counteract ROS-related damage. Proteomic analysis evidenced 60 differentially abundant spots, of which 58 were identified by mass spectrometry, 24 up- and 36 down-regulated, as reported in Figure S3 and Table S2. PCA in Figure 4A shows the distribution of Dsup− and Dsup+ samples along the two principal components, which together represent 97.18% of the variance of the %V of differential spots. According to the PCA, the Dsup+ proteomic profile well differentiates from that of Dsup− cells, evidencing the Dsup impact in modulating the response of human cells to oxidative stress. Particularly, the increased abundance of ubiquitin carboxyl-terminal hydrolase isozyme L3 (UCHL3), thioredoxin (THIO), and 60 S acidic ribosomal protein P0 (RLA0), and the decreased abundance of two protein species of polyubiquitin-B (UBB) have a greater relevance in discriminating Dsup−/+ cells. Based on heatmap analysis (Figure 4B), differential spots similarly behave in terms of abundance within the same condition, leading Dsup− and Dsup+ samples to cluster well into two distinct groups. On the other hand, the heatmap clearly highlights two main clusters of spots (rows) that oppositely behave in Dsup+ cells with respect to the Dsup− cells: one with 36 protein spots that are less abundant in Dsup+ cells and one with 24 protein spots that are highly abundant in Dsup+ cells. Considering these two main clusters of protein spots, an enrichment analysis by GO biological processes (BPs) was performed. The most represented BPs associated with the up-regulated proteins in Dsup+ cells after oxidative stress are protein folding, glycolytic processes, mRNA splicing via the spliceosome, cell-redox homeostasis, regulation of macro-autophagy, and post-translational protein modifications. On the other hand, the most represented BPs associated with the down-regulated proteins in Dsup+ cells after oxidative stress are anaphase-promoting complex dependent catabolic processes, pre-replicative complex assembly, regulation of cellular amino acid metabolic processes, protein refolding, antigen processing and presentation via MHC class I, TAP-dependent regulation of transcription from RNA polymerase II promoter in response to hypoxia, negative regulation of G2/M transition of mitotic cell cycle, ATP metabolic process, regulation of mRNA stability, post-translational protein modification, and proteasome-mediated ubiquitin-dependent protein catabolic processes (Figure 4C).

### 2.4. Confirmation of the Identified Targets

**Figure 5:**
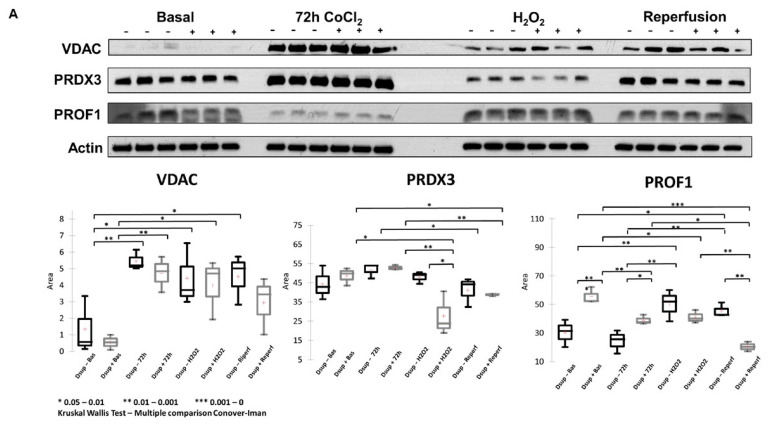

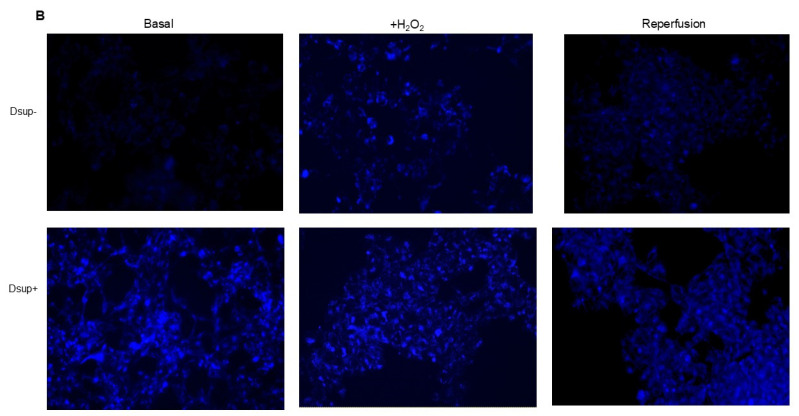
(**A**) Western blot validations. Figure reports WB validations of VDAC, PRDX3, and PROF1, and normalization of bands’ intensity was performed on total β-actin. The Kruskal–Wallis test was performed for each WB validation, as reported by box plots. Significant variation in intensity is reported as * when the *p*-value is in the range 0.05 and 0.01, ** when the *p*-value is in the range 0.0 and 0.001, and *** when the *p*-value is lower than 0.001. (**B**) Autophagy in Dsup− or Dsup+ cells at basal conditions or treated with 250 µM H_2_O_2_ O/N or the reperfusion protocol. Fluorescence intensity (lex = 360/lem = 520 nm) was measured using a fluorescence microscope (Nikon Eclipse Ts2). Autophagy was indicated by bright blue dot staining of autophagic vacuoles. Images are taken at 20X.

Mitochondrial activity has a central role in response to hypoxia as well as oxidative stress. Treatment with CoCl_2_ highlighted proteins involved in insertion into the mitochondrial membrane, such as voltage-dependent anion channel (VDAC) proteins VDAC1 and VDAC2, which we found dysregulated, by proteomic analysis, after hypoxic stress and after reperfusion (Table S1). VDAC1 and VDAC2 have roles in pore-forming proteins and are known to play an essential role in cellular metabolism and in the early stages of apoptosis. For example, VDAC constitutes a major pathway by which metabolites such as ADP/ATP, succinate, and citrate are exchanged between the cytosol and mitochondria. VDAC1 in the plasma membrane establishes a novel level of apoptosis regulation, putatively via its redox activity. Western blot analysis of VDAC after CoCl_2_ and H_2_O_2,_ and reperfusion showed VDAC increased after CoCl_2_ and H_2_O_2_ treatment and after reperfusion in both Dsup+/−, with respect to the basal condition (Figure 5A). Of note, after reperfusion, in Dsup+ cells, there was not a significant increase in VDAC, indicating that in these cells, a lower stress condition is maintained.

Our proteomic data showed dysregulation of PRDX1, PRDX3, and PRDX6, reflecting a regulation of peroxidase activity after hypoxic insult and cell redox homeostasis regulation after oxidative stress induced by H_2_O_2_. By Western blot analysis, we analyzed the levels of PRDX3, a thiol-specific peroxidase that catalyzes the reduction of hydrogen peroxide and organic hydroperoxides to water and alcohols, playing a role in cell protection against oxidative stress. PRDX3 levels did not change in abundance in Dsup+/− cells in the presence of hypoxia (CoCl_2_) with respect to the basal condition. Its different behavior (downregulation) is observed in Dsup+ cells after H_2_O_2_ treatment. Similarly, PRDX3 tends to be downregulated also in Dsup+ cells after reperfusion (Figure 5A).

Another interesting protein, highlighted by differential proteomic analysis among Dsup+/− cells after 72 h of hypoxic stress, subsequent reperfusion, is profilin 1. This is an actin-binding protein involved in the dynamic transformation and reorganization of actin. Its validation by Western blot showed PROF-1 upregulation in Dsup+ cells at basal conditions and after CoCl_2_ treatment for 72 h. On the other hand, PROF-1 levels decrease in Dsup+ cells after H_2_O_2_ treatment and overall, after reperfusion, suggesting an alternative role in the presence of oxidative stress with respect to hypoxic stress.

Among up-regulated proteins in Dsup+ cells following reperfusion and H_2_O_2_ treatment, we found those involved in autophagy mechanisms, whereas the same proteins are down-regulated in Dsup+ cells after CoCl_2_ treatment. Immunohistochemical analysis clearly shows (Figure 5B) that Dsup presence induces an increase in autophagy both at baseline and following H_2_O_2_ treatment and reperfusion compared to untransfected cells.

## 3. Discussion

Ischemia–reperfusion injury (IRI) is a significant clinical challenge, characterized by a paradoxical exacerbation of cellular damage when blood flow is restored to ischemic tissue. A primary driver of this damage is the substantial production of reactive oxygen species (ROS) during reperfusion, which overwhelms the cell’s antioxidant defenses and triggers a series of events that lead to cell death. The endoplasmic reticulum (ER) stress pathway is activated in damaged and hypoxic cells due to an increased amount of damaged or misfolded proteins and the accumulation of free fatty acids and other lipids resulting from an inability to oxidize them (lipotoxicity) [22].

The present study investigated the ability of the tardigrade-derived damage suppressor (Dsup) protein to protect human cells from IRI, using an in vitro model of hypoxia and reoxygenation. Our findings show that the presence of the Dsup increases the survival rate of HEK293TT cells exposed to hypoxia and induces the expression of genes associated with the hypoxia response. It up-regulates proteins involved in the negative regulation of the stress-activated MAPK cascade, neutrophil aggregation, peroxidase activity, nitric oxide storage, and several biosynthetic and metabolic processes. Conversely, it down-regulates proteins associated with the organization of the outer mitochondrial membrane, chaperone-mediated protein complex assembly, the positive regulation of protein polymerization, chaperone-mediated autophagy, and the organization of organelles and chromosomes.

Furthermore, our study provides compelling evidence that the Dsup confers significant cytoprotection after simulated reperfusion, primarily by mitigating oxidative stress and modulating key pathways involved in the cellular stress response and protein homeostasis. A key finding is that Dsup-expressing HEK293TT cells (Dsup+) have enhanced viability compared to control cells (Dsup−) following hypoxic stress and reperfusion. This protective effect is directly linked to the antioxidant properties of the Dsup, as demonstrated by the significantly lower levels of ROS and a decreased GSH/GSSG ratio in Dsup+ cells during the reperfusion phase. These results are in line with the initial characterization of the Dsup, which demonstrated its ability to protect DNA from oxidative damage [23].

Our study expands on this by showing that the Dsup protects cells from IRI not just by defending the DNA but also by generally reducing oxidative stress.

To deepen the molecular mechanisms underlying the Dsup’s protective role, we performed a comparative proteomic analysis of Dsup+ and Dsup− cells. This analysis revealed that Dsup expression leads to the dysregulation of several proteins involved in the endogenous antioxidant response, including peroxiredoxins (PRDX1, 3, and PRDX6). Peroxiredoxins are a ubiquitous family of antioxidant enzymes that catalyze the reduction of hydrogen peroxide and other organic hydroperoxides, playing a crucial role in cellular protection against oxidative stress [24]. Specifically, PRDX1 is a key cytosolic antioxidant and one of the most efficient enzymes for detoxifying H_2_O_2_ [25]. The Dsup effectively enhances the cell’s primary defense against oxidative stress by regulating the levels of PRDX1, directly lowering the concentration of damaging ROS in the cytosol. This prevents downstream damage to proteins, lipids, and DNA. On the other hand, PRDX6 assists in repairing cell membranes damaged by lipid peroxidation [26], working in concert with PRDX1 to promote the restoration of compromised cellular structures. PRDX3, an efficient H_2_O_2_ scavenger, protects the cells from mitochondrial oxidative damage and apoptosis [27]. Interestingly, after IRI and after the H_2_O_2_ stimulus, it remains lower than in the Dsup− counterpart, suggesting that cells transfected with the Dsup experience less stress, as also suggested by the behavior of PRDX1 and PRDX6. The modulation of these proteins in Dsup+ cells is significant, not only because it enhances antioxidant defenses directly, but also because of its interaction with the hypoxia signaling pathway. Peroxiredoxins impair HIF-1 and HIF-2 binding to the hypoxia response elements of a subset of HIF target genes, thereby inhibiting gene transcription in cells exposed to prolonged hypoxia [24]. Therefore, the modulation of peroxiredoxins observed in Dsup+ cells may provide greater protection against reperfusion-induced oxidative damage and contribute to more effective and controlled management of the transcriptional response to hypoxia. This limits the potentially harmful effects of prolonged HIF activation.

Our proteomic analysis revealed an enrichment of proteins associated with the negative regulation of the stress-activated MAPK cascade in Dsup-expressing cells. This suggests that the Dsup may inhibit proinflammatory and apoptotic signaling pathways, which are usually activated by cellular stress. This anti-inflammatory effect is further supported by the upregulation of proteins that negatively regulate neutrophil aggregation, a key event in reperfusion injury where the accumulation of neutrophils exacerbates tissue damage [28]. The upregulation of proteins related to nitric oxide storage suggests a mechanism for buffering and controlling the release of NO, which could prevent its reaction with superoxide to form the highly damaging peroxynitrite radical. We also noted an increase in proteins that govern the metabolic processes of pyruvate and monocarboxylic acids. This probably indicates an enhanced capacity to manage pyruvate, the central hub of energy metabolism, and to process monocarboxylic acids such as lactate, which is the main byproduct of anaerobic glycolysis during ischemia [29]. This metabolic flexibility is crucial for sustaining energy production and mitigating the damaging effects of lactic acidosis. Although the increased production of methylglyoxal and aldehydes may initially seem unfavorable, it could indicate a metabolic shunt or a preconditioning response that prepares the cell to detoxify these reactive carbonyl species more efficiently when it is reoxygenated. Similarly, proteins involved in the negative regulation of peroxidase activity could reflect a feedback mechanism whereby the Dsup’s primary antioxidant activity is so effective that the cell reduces its own enzymatic systems in order to maintain redox homeostasis.

Another important aspect of Dsup’s role in cell protection is its modulation of proteins involved in the reorganization and insertion of the mitochondrial outer membrane. IRI processes lead to pathological signals converging on the mitochondria and inducing their membrane, particularly through mechanisms involving permeabilization of the outer membrane. This ultimately leads to cell death [30]. Therefore, any strategy that can limit mitochondrial membrane permeabilization has the potential to be protective. In Dsup+ cells, modulation of VDAC proteins could be helpful in this respect.

Among the pathways that counteract the negative consequences of ischemia–reperfusion, autophagy is also involved. Autophagy removes damaged organelles and molecules, driving them to lysosomes where they are digested into simpler compounds that then become an energy source for the cell. Mitophagy and ER-phagy result in improvement of cell energetic balance and alleviation of ER stress [22]. Interestingly, among the differentially abundant proteins we found, profilin-1 showed a different behavior between hypoxic and oxidative insults. Recently, profilin-1 was reported to have different interesting roles that cannot be explained by its primary function as a cytosolic actin assembly factor, as it was seen to be involved in maintaining mitochondrial integrity and in mitophagy [31]. The downregulation of profilin-1 in Dsup+ cells after oxidative stress and during reperfusion overall suggests an advantage in restoring cellular abilities, as was also observed by Lu et al. [32]. They performed the knockdown of profilin-1 and observed a significant attenuation of brain infarcts and edema.

It is worth noting that an increase in autophagy is also evident under basal conditions in comparison to Dsup− cells. Previous studies have shown that cells expressing the Dsup undergo certain changes even in the absence of external stimuli. For example, changes in the expression of several genes and proteins, mainly those involved in DNA damage and repair, have been reported in Dsup-expressing plants, HEK293T cells, and D. melanogaster under normal conditions, suggesting that DNA repair pathways are activated prior to damage [21,33,34]. The question of whether these changes are beneficial to the cell, preparing it for potential damage, or whether they are a cellular response to a stressful condition related to the presence of a heterologous protein remains unanswered. In the case of increased autophagy, a cellular response to a heterologous protein may be hypothesized. However, in this way, the Dsup may help to maintain protein homeostasis and prevent the activation of apoptotic pathways by promoting the efficient clearance of damaged proteins. This indicates that the Dsup plays a more intricate role in cellular maintenance and survival than was previously thought.

The main strength of this study lies in our multi-approach, which combines functional assays with a comprehensive proteomic analysis to examine the mechanisms of Dsup-mediated protection. This has enabled us to progress from a basic description of the Dsup’s protective effects to a more detailed understanding of its function. We know that other studies have demonstrated the protective effects of the Dsup against general oxidative stress [19,20]. However, we would like to emphasize that, to our knowledge, this is the first study to investigate the role and cytoprotective mechanisms of the Dsup specifically under conditions of hypoxia. The experiments using H_2_O_2_ were included to simulate aspects of the IRI environment, but the central focus of our paper is the function of the Dsup protein in a human cellular environment under hypoxic stress. In our research, by subjecting Dsup-expressing human cells to a hypoxic environment, we are probing the protein’s function in a cellular environment that is central to the pathophysiology of major clinical conditions, including ischemia–reperfusion injury, cancer, and stroke. The distinction between these two stressors is critical: applying an external agent like H_2_O_2_ induces direct, widespread, and somewhat indiscriminate oxidative damage. In contrast, hypoxia triggers a specific, highly regulated, and adaptive cellular program orchestrated by the Hypoxia-Inducible Factor (HIF) signaling pathway. This pathway leads to a profound reprogramming of cellular metabolism and gene expression. Consequently, by testing the Dsup’s function under hypoxia, we are not simply observing its effect against generalized damage but rather investigating its role within a distinct biological signaling pathway. This exploration of the Dsup in the context of a specific adaptive response, rather than just a damage response, is what constitutes the novelty and significance of our findings.

However, our study is not without limitations. The use of a single cell line, although HEK293T cells are a widely recognized tool for transfection studies, may not fully illustrate the complex pathophysiology of IRI in specific tissues, such as the heart or brain. Future studies should therefore aim to validate these findings in more clinically relevant cell types, such as cardiomyocytes or neuronal cells, or animal models of IRI. Additionally, while our study provides strong evidence of the involvement of several pathways, the precise molecular interactions between the Dsup and these pathways still need to be elucidated. Finally, as this is an in vitro study, the therapeutic potential of the Dsup in a whole-organism context has yet to be demonstrated.

## 4. Materials and Methods

### 4.1. Cell Cultures and Dsup Transfection

The HEK293TT cell line (mycoplasma-free, verified by N-GARDE Mycoplasma PCR reagent set, Euroclone, Milan, Italy) was kindly donated by Prof. Sandra Donnini (University of Siena). pCXN2KS-Dsup was a gift from Prof. Kunieda Takekazu (Addgene plasmid #90019; https://www.addgene.org/90019/ RRID: Addgene_90019 accessed on 20 February 2021). Cells were cultured in Dulbecco’s modified Eagle’s Medium (DMEM) supplemented with 10% fetal bovine serum (FBS). An empty vector (plasmid without Dsup) was kindly donated by Dr. Jlenia Brunetti (University of Siena). Both constructs were transfected into HEK293TT cells using Lipofectamine^®^ 2000 Reagent (Life Technologies, Milna, Italy) and selected by 700 µg/mL G418 (Euroclone) for three weeks. Transfection was verified by endpoint PCR as already reported [20].

### 4.2. Cell Viability

The MTT metabolic assay (Vybrant^®^ MTT Cell Proliferation Assay Kit, Molecular Probes, Eugene, OR, USA) was used to quantify cell viability. Both Dsup+ and empty vector cells (Dsup−) were seeded at a 100,000 cells/mL density in a 96-well plate. After 24 h of incubation, to induce hypoxia, cells were treated for 24, 48, and 72 h with 100 μM CoCl_2_ [35]. To simulate reperfusion, we allowed cells to grow in normal medium for 24 h after 72 h of treatment with CoCl_2_. To induce oxidative stress, cells were treated with 250 µM hydrogen peroxide (H_2_O_2_) O/N. After each stimulation, cells were incubated at 37 °C for 4 h with the tetrazolium dye MTT (3-(4,5-Dimethylthiazol-2-yl)-2,5-Diphenyltetrazolium Bromide). Solubilization was carried out with DMSO (50 µL) at 37 °C for 10 min. Then, the number of viable cells was determined by measuring absorbance at 540 nm in a microplate reader (Tecan). Each experiment was run in triplicate and was repeated at least three times.

### 4.3. Real Time qPCR

Total RNA was extracted from cell pellets using the SV Total RNA Isolation System (Promega, Milan, Italy) following the manufacturer’s instructions. The TaqMan™ Array Human Hypoxia (ThermoFisher Scientific, Milan, Italy) was run on 100 ng of cDNA obtained by retrotranscription of RNA with the iScript cDNA Synthesis kit (Biorad, Milan, Italy). The hypoxia assay contains 44 hypoxia-related assays and 4 assays to candidate endogenous control genes. The assay was plated in duplicate. Quantification was determined by using the 2−ΔCT method.

### 4.4. Proteomic Analysis

Three independent cell cultures per condition, as biological replicates, in both cells transfected with Dsup (Dsup+) and cells transfected with an empty vector (Dsup−) were used for proteomic analyses. Three main conditions were analyzed, such as oxidative stress by H_2_O_2_ stimulation, 72 h-hypoxia by CoCl_2_ stimulation, and reperfusion by restoring normal O_2_ conditions.

Cell cultures were collected by trypsinization and washed with phosphate-buffered saline (PBS) three times, followed by a centrifugation of 5 min at 1600× *g* after each wash. Cell pellets were kept at −80 °C until use.

Samples were dissolved in a lysis buffer composed of 7 M urea, 2 M thiourea, 4% *w*/*v* 3-[(3-cholamidopropyl) dimethylammonia]-1-propanesulfonate hydrate (CHAPS), and 1% *w*/*v* dithioerythritol (DTE). After having determined the concentration of the proteins contained in the samples by Bradford assay, each sample was resolved following the two-dimensional electrophoresis (2DE) technique [36]. Image analysis was then performed by Melanie™ Classic 9 software (SIB Swiss Institute of Bioinformatics, Geneva, Switzerland), which allowed the determination of the percentage of relative volume (%V) of each spot. Based on these values, a statistical analysis was performed to identify statistically significant differential spots by ANOVA test via XLStat (Addinsoft, Paris, France), according to *p*-value ≤ 0.05 and fold change ≥ 1.8 for H_2_O_2_ analysis, and a fold change ≥ 2 for hypoxia/reperfusion analysis.

### 4.5. Protein Identification by MALDI-ToF Mass Spectrometry

The identification of the differential spots was performed by MALDI-ToF mass spectrometry through peptide mass fingerprinting (PMF). Differential spots were detected in the MS-compatible silver-stained gels and manually excised from them. To avoid possible interferences due to the staining, spots were destained first in a solution of 30 mM potassium ferricyanide and 100 mM anhydrous sodium sulfate, and then in 200 mM ammonium bicarbonate. Then, they went through a dehydration process in 100% acetonitrile (ACN). After the following rehydration, protein spots were digested in a trypsin solution overnight at 37 °C, then placed on a MALDI target and covered with a matrix solution of 5 mg/mL α-cyano-4-hydroxycinnamic acid (CHCA) in 50% *v*/*v* ACN and 0.5% *v*/*v* trifluoroacetic acid (TFA). The UltrafleXtreme™ MALDI-ToF/ToF mass spectrometer (Bruker Daltoniks, Bremen, Germany), equipped with a 200 Hz smartbeam™ I laser in the positive reflector mode, was used to carry out the mass spectrometry analysis with the following parameters: 170 ns of delay; ion source 1:20 kV; ion source 2:17.70 kV; lens voltage: 7.80 kV; reflector voltage: 20.80 kV; and reflector 2 voltage: 10.95 kV. The applied laser wavelength and frequency were 353 nm and 100 Hz, respectively, and the percentage was set to 50%. MS spectra were obtained considering the averages of 1500 laser shots targeted at five different positions within the spot. The acquisition of mass spectra was carried out by FleXcontrol software v 3.0 (Bruker), and the processing was carried out via the FleXanalysis software version 3.0 (Bruker), considering the peptides obtained from the trypsin autoproteolysis as internal standards for calibration. In the resulting mass lists, common contaminants (such as matrix-related ions, trypsin autolysis, and keratin peaks) were pinpointed and excluded. The identification of the protein spots was performed using the PMF search with MASCOT (Matrix Science Ltd., London, UK, http://www.matrixscience.com; accessed on 15 March 2024). The following parameters were set up: Homo sapiens as taxonomy, SwissProt as database, 20 ppm as mass tolerance, one admissible missed cleavage site, carbamidomethylation (iodoacetamide alkylation) of cysteine as a fixed modification, and oxidation of methionine as a variable modification. The following inclusion criteria were considered for the protein identifications: a *p*-value < 0.02 (referred to as “expect”, as reported in Tables S1 and S2), a minimum of three matched peptides, and a minimum MASCOT score of 55.

The mass spectrometry proteomics data have been deposited to the ProteomeXchange Consortium the PRIDE [37] partner repository with the dataset identifier PXD067125.

### 4.6. Western Blot

Twenty-five micrograms of three different biological replicates per condition were suspended in LEAMMLI buffer (Tris HCl pH 6.8, 62.5 mM, 20% *v*/*v* glycerol, 2% *w*/*v* SDS, 5% *v*/*v* β-mercaptoethanol, and 0.032% *w*/*v* bromophenol blue), and samples were heated at 95 °C for 7 min. After cooling, samples were loaded in 12% polyacrylamide gels. The Mini-PROTEAN electrophoresis system (Bio-Rad, Heracles, CA, USA) was used to carry out mono-dimensional electrophoresis. After SDS-PAGE, the 1 h equilibration of gels took place in the transfer buffer (192 mM glycine, 25 mM Tris, 20% *v*/*v* methanol), and then, gels were transferred to nitrocellulose membranes (Cytiva, Washington, DC, USA). To evaluate if the protein transfer had correctly taken place, membranes were stained with Red Ponceau (0.2% *w*/*v* Ponceau S in 3% acetic acid).

Membranes were tested for three different primary antibodies, such as anti-VDAC (rabbit polyclonal, PA1-954, Invitrogen, Waltham, MA, USA, WB dilution 2 μg/mL), anti-PROF1 (rabbit polyclonal, P7624, Sigma-Aldrich, St. Louis, MO, USA, WB dilution 2 μg/mL), and anti-PRDX3 (mouse monoclonal, sc-59663, Santa Cruz Biotechnology, Dallas, TX, USA, dilution 1:200). Secondary antibody incubation varies according to primary antibody composition; in detail, secondary peroxidase-conjugated goat anti-rabbit antibody was used at 1:7000 dilution and secondary peroxidase-conjugated goat anti-mouse antibody at 1:3000 dilution. Western blot normalization was performed on total β-actin (anti-β-actin mouse monoclonal, A5441, Sigma, NY, USA, dilution: 1:40,000). Immunoreaction was detected by a chemiluminescence detection system (Cytiva), and images were analyzed with ImageJ v 1.x (1.51p, NIH, Chicago, IL, USA) to obtain band intensity. Statistical analysis was performed by XLSTAT (Addinsoft).

### 4.7. Autophagy Assay

To assess autophagy, 5 × 104 cells were plated in 24-well plates and allowed to grow for 24 h. Cells were then stimulated O/N with 250 µM H_2_O_2_ or with the hypoxia/reperfusion protocol (72 h of 100 μM CoCl_2_ and 24 h of normal medium). Autophagy was assessed using the Autophagy Assay Kit (Sigma-Aldrich, NY, USA) following the kit instructions. Briefly, the medium was removed, and 100 µL of the Autophagosome Detection Reagent was added to each well. Cells were incubated at 37 °C with 5% CO_2_ for 1 h. After incubation, cells were washed 4 times with the wash buffer by gently adding 100 μL of wash buffer to each well. Fluorescence intensity (lex = 360/lem = 520 nm) was measured using a fluorescence microscope (Nikon Eclipse Ts2). Autophagy was indicated by bright blue dot staining of autophagic vacuoles.

### 4.8. Oxidative Stress Level Measurement

The ROS-Glo™ H_2_O_2_ Assay (Promega) was used to measure the level of H_2_O_2_ directly in the cell culture. Briefly, 1.5 × 103 cells were plated in 96-well plates and treated with the hypoxia/reperfusion protocol (72 h of 100 μM CoCl_2_ and 24 h of normal medium). According to the kit instructions, 20 µL of H_2_O_2_ Substrate Solution was incubated at 37 °C for the last 6 h. This reacts directly with H_2_O_2_ to generate a luciferin precursor. Then, 100 µL of the ROS-Glo™ Detection Solution was added for 20 min to convert the precursor to luciferin. The light signal is proportional to the level of H_2_O_2_ present in each sample.

The GSH/GSSG-Glo™ Assay (Promega) was used to measure the ratio of GSH/GSSG. Briefly, 5 × 103 cells were plated in 96-well plates and treated with the hypoxia/reperfusion protocol. At the end of reperfusion, we removed the medium and performed a wash with PBS to remove any residue of cell culture media that may interfere with the assay (as reported in the protocol). Then, according to the kit instruction, 50 µL of Total Glutathione Lysis Reagent (for GSH + GSSG wells) or Oxidized Glutathione Lysis Reagent (for GSSG wells) was added. Total Glutathione Lysis Reagent lyses cells and reduces GSSG to GSH to measure total glutathione. Oxidized Glutathione Lysis Reagent lyses cells and blocks the GSH present in the sample to produce a form that cannot contribute to the luminescent signal, then it converts GSSG into new GSH. In both formulations, the luciferin-NT is converted to luciferin in a GSH-dependent reaction, which is coupled to a firefly luciferase reaction. The amount of light emitted by luciferase depends on the amount of luciferin formed, which in turn depends on the amount of GSH present. The plate was placed on a plate shaker for 5 min, and 50 µL of Luciferin Generation Reagent was added to each well for 30 min (room temperature). Finally, 100 µL of Luciferin Detection Reagent was added to each well to block the luciferin generation reaction and initiate a luminescent signal.

### 4.9. Statistical Analysis and Enrichment Analyses

GraphPad Prism software version 5 was used for statistical analysis. For qPCR analysis, at least three separate replicates were performed. Sample differences were assessed by a one-way ANOVA test. Survival data were analyzed using a paired test for non-parametric data (Wilcoxon signed-rank test). Principal Component Analysis (PCA) and heatmap analysis were performed by XLSTAT for both H_2_O_2_ and hypoxia/reperfusion proteomic analyses, comparing Dsup− and Dsup+ cells, considering the %V of statistically significant differential protein spots. PCA was carried out Pearson’s correlation and heatmap analysis using the Euclidean distance.

Enrichment analyses were carried out using the DAVID Bioinformatics platform and MetaCore software (https://portal.genego.com/, accessed in 7 April 2024) (Clarivate Analytics, Boston, MA, USA). Enrichment by Gene Ontology (GO) was performed for cellular compartments and biological processes, as well as enrichment by pathway maps and process networks.

Results from Western blot were analyzed by the Kruskal–Wallis test with the multiple comparison Conover-Iman. For all comparisons, a *p* value of <0.05 was considered significant (* < 0.05; ** < 0.01; *** < 0.001).

## 5. Conclusions

In conclusion, this study provides a significant advancement in our understanding of the protective mechanisms of the tardigrade protein Dsup. We have demonstrated that the Dsup protects human cells from ischemia–reperfusion injury by mitigating oxidative stress, enhancing endogenous antioxidant defenses, and modulating key pathways involved in protein homeostasis and cellular stress response. The remarkable protective properties of the Dsup to withstand extreme environments hold great promise for translation into clinical applications to protect human cells from the damaging consequences of oxidative stress. Further in vivo studies are warranted to fully explore the therapeutic potential of this unique protein.

## Figures and Tables

**Table 1:** Differentially expressed hypoxia-related genes in Dsup+ cells after 72 h of 100 µM CoCl_2_.

Gene	2^-dctt Fold Change
*ADM*	0.70
*ANGPTL4*	no amplification
*ARNT*	1.95
*ARNT2*	1.55
*ATP1B1*	1.51
*BHLHE40*	1.13
*CASP1*	no amplification
*CREBBP*	1.16
*DDIT4*	1.27
*DDIT4L*	1.06
*EDN1*	0.83
*EGLN1*	1.22
*EGLN2*	0.72
*EGLN3*	0.94
*EP300*	0.99
*EPAS1*	1.23
*EPO*	no amplification
*FRAP1*	1.11
*HIF1A*	1.64
*HIF1AN*	1.09
*HIF3A*	no amplification
*HIG2*	0.81
*HMOX1*	1.62
*HYOU1*	1.57
*IGFBP1*	no amplification
*ING4*	0.65
*MB*	no amplification
*MT3*	no amplification
*NOS1*	1.37
*NOS2*	1.68
*NOS3*	no amplification
*NOTCH1*	1.91
*PIK3CA*	1.42
*PRKAA1*	0.99
*PRKAA2*	1.13
*PTEN*	0.64
*SLC2A8*	1.21
*SOD3*	no amplification
*TGFBR2*	0.62
*TP53*	1.37
*VEGFA*	0.61
*VHL*	1.68
*CUL2*	1.69
*RBX1*	0.84

## Data Availability

The mass spectrometry proteomics data have been deposited to the Proteo-meXchange Consortium Via the PRIDE partner repository with the dataset identifier PXD067125.

## Supplementary Materials

The following supporting information can be downloaded at: https://www.mdpi.com/article/10.3390/ijms262110452/s1.
