# Supplementary material for: Investigating the Cytoprotective Mechanisms of the Tardigrade Damage Suppressor (Dsup) Protein in Human Cells Under Hypoxic Stress
Source: Int J Mol Sci. 2025 Oct 28;26(21):10452. doi: 10.3390/ijms262110452 (PMC12607402; doi:10.3390/ijms262110452)
Supplement: Supplementary file 1 [file ijms-26-10452-s001.zip › Hypoxia supplementary tables.pdf]

Table S1: Proteomic data and identifications of differential spots in Dsup-/+ HEK293T cells after 72h of hypoxic stress with $CoCl_2$ and following 24h recovery after restoring normal O_2 conditions

Table reports the spot numbers corresponding to that in Figure S3, protein name when the protein was identified by MALDI-ToF MS, UniProt Entry name, accession number (AC), and gene name, the ANOVA Test and the mean of the %V of the specific spot in cells after 72h of hypoxic stress and following 24h recovery after restoring normal O_2 levels (Reperfusion), respectively in Dsup- and Dsup+ cells. The right part of the table is dedicated to Mascot Search Results such as pI and MW, Expect and Score, Matched Peptides and sequence Coverage (%). Only a differential abundance (Fold Change) ≥ 2 was considered for each spot. Letters "a, b, c, d" are used to mark significative differential abundance in the following comparisons, respectively: "a" for Dsup-/+ 72h, "b" for Dsup-/+ Reperfusion, "c" Dsup- 72h *vs* Reperfusion, "d" Dsup+ 72h *vs* Reperfusion.

<table>
<tr><th rowspan="2">Spot n°</th><th rowspan="2">Protein name</th><th rowspan="2">UniProt Name</th><th rowspan="2">AC</th><th rowspan="2">Gene Name</th><th colspan="5">Anova test</th><th colspan="6">Mascot Search Results</th></tr>
<tr><th>Anova (p)</th><th>Dsup- (72h)</th><th>Dsup+ (72h)</th><th>Dsup- (Reperf)</th><th>Dsup+ (Reperf)</th><th>pI - MW</th><th>Expect</th><th colspan="2">Score</th><th>Matched peptides</th><th>Coverage (%)</th></tr>
<tr><td>1</td><td>Heat shock 70 kDa protein 4</td><td>HSP74_HUMAN</td><td>P34932</td><td>HSPA4</td><td>0,0100 [a d]</td><td>0,02657</td><td>0,01021</td><td>0,01937</td><td>0,02631</td><td>5,11 - 95127</td><td>5.1e-09</td><td colspan="2">126</td><td>11/15</td><td>18</td></tr>
<tr><td>2</td><td>Phosphoribosylformylglycinamidine synthase</td><td>PUR4_HUMAN</td><td>O15067</td><td>PFAS</td><td>0,0193 [a]</td><td>0,01967</td><td>0,00726</td><td>0,00988</td><td>0,00724</td><td>5,50 - 146297</td><td>1.6e-11</td><td colspan="2">151</td><td>16/27</td><td>20</td></tr>
<tr><td>3</td><td>Matrin-3</td><td>MATR3_HUMAN</td><td>P43243</td><td>MATR3</td><td>0,0029 [a d]</td><td>0,01652</td><td>0,00445</td><td>0,01560</td><td>0,01861</td><td>5,87 - 95078</td><td>2.6e-17</td><td colspan="2">209</td><td>19/25</td><td>28</td></tr>
<tr><td>4</td><td>Heat shock protein HSP 90-alpha</td><td>HS90A_HUMAN</td><td>P07900</td><td>HSP90AA1</td><td>0,0325 [a d]</td><td>0,02148</td><td>0,00587</td><td>0,01505</td><td>0,02284</td><td>4,94 - 85006</td><td>3.2e-14</td><td colspan="2">178</td><td>17/24</td><td>30</td></tr>
<tr><td>5</td><td>Heat shock 70 kDa protein 4</td><td>HSP74_HUMAN</td><td>P34932</td><td>HSPA4</td><td>0,0127 [a c]</td><td>0,02905</td><td>0,00994</td><td>0,01366</td><td>0,02463</td><td>5,11 - 95127</td><td>1.6e-32</td><td colspan="2">361</td><td>31/36</td><td>44</td></tr>
<tr><td>6</td><td>Ubiquitin-like modifier-activating enzyme 1</td><td>UBA1_HUMAN</td><td>P22314</td><td>UBA1</td><td>0,0114 [a b]</td><td>0,00846</td><td>0,00393</td><td>0,01128</td><td>0,00324</td><td>5,49 - 118858</td><td>2.6e-21</td><td colspan="2">249</td><td>20/24</td><td>29</td></tr>
<tr><td>7</td><td></td><td></td><td></td><td></td><td>0,0008 [b c d]</td><td>0,00169</td><td>0,00116</td><td>0,01037</td><td>0,00429</td><td></td><td></td><td colspan="2"></td><td></td><td></td></tr>
<tr><td>8</td><td>Alpha-actinin-4</td><td>ACTN4_HUMAN</td><td>O43707</td><td>ACTN4</td><td>0,0114 [a d]</td><td>0,02457</td><td>0,01069</td><td>0,01512</td><td>0,03020</td><td>5,27 - 105245</td><td>2.6e-28</td><td colspan="2">319</td><td>31/45</td><td>40</td></tr>
<tr><td rowspan="3">9</td><td>Ubiquitin-like modifier-activating enzyme 1</td><td>UBA1_HUMAN</td><td>P22314</td><td>UBA1</td><td rowspan="3">0,0375 [a b d]</td><td rowspan="3">0,02453</td><td rowspan="3">0,01135</td><td rowspan="3">0,01583</td><td rowspan="3">0,03592</td><td>5,49 - 118858</td><td>0.003</td><td rowspan="3">213</td><td>68</td><td>11/42</td><td>17</td></tr>
<tr><td>DNA replication licensing factor MCM6</td><td>MCM6_HUMAN</td><td>Q14566</td><td>MCM6</td><td>5,29 - 93801</td><td>0.00087</td><td>74</td><td>11/42</td><td>19</td></tr>
<tr><td>Alpha-actinin-4</td><td>ACTN4_HUMAN</td><td>O43707</td><td>ACTN4</td><td>5,27 - 105245</td><td>1.3e-07</td><td>112</td><td>15/42</td><td>23</td></tr>
<tr><td>10</td><td>DNA replication licensing factor MCM4</td><td>MCM4_HUMAN</td><td>P33991</td><td>MCM4</td><td>0,0307 [a]</td><td>0,02624</td><td>0,00799</td><td>0,01447</td><td>0,01119</td><td>6,28 - 97068</td><td>1,00e-09</td><td colspan="2">133</td><td>11/13</td><td>16</td></tr>
</table>

**11**	**Methionine--tRNA ligase, cytoplasmic**	**SYMC_HUMAN**	**P56192**	**MARS1**	**0,0290** [a d]	0,01536	0,00492	0,02470	0,02289	5,82 - 102249	2.6e-24	279		26/35	34
**12**	**Glucosidase 2 subunit beta**	**GLU2B_HUMAN**	**P14314**	**PRKCSH**	**0,0078** [c d]	0,17765	0,10462	0,04702	0,02623	4,33 - 60357	5.1e-13	166		12/16	25
**13**					**0,0189** [c d]	0,12259	0,07723	0,01299	0,01365						
**14**					**0,0302** [c]	0,14577	0,09989	0,02157	0,05829						
**15**					**0,0433** [c d]	0,05677	0,04039	0,01433	0,00894						
**16**	**Far upstream element-binding protein 1**	**FUBP1_HUMAN**	**Q96AE4**	**FUBP1**	**0,0018** [b]	0,01702	0,02237	0,02130	0,00866	7,18 - 67690	8.1e-14	174		13/18	30
**17**	**Far upstream element-binding protein 1**	**FUBP1_HUMAN**	**Q96AE4**	**FUBP1**	**0,0052** [b]	0,04350	0,04533	0,03337	0,01480	7,18 - 67690	8.1e-17	204		17/25	36
**18**	**Ubiquitin-like modifier-activating enzyme 1** ***C-term fragment***	**UBA1_HUMAN**	**P22314**	**UBA1**	**0,0025** [c d]	0,01043	0,00803	0,04503	0,07575	5,49 - 118858	1,00e-26	303		29/41	37
**19**	**60 kDa heat shock protein, mitochondrial**	**CH60_HUMAN**	**P10809**	**HSPD1**	**0,0033** [b c]	0,19304	0,10583	0,07353	0,14754	5,70 - 61187	2.6e-14	179		18/41	45
**21**	**Telomeric repeat-binding factor 2-interacting protein 1**	**TE2IP_HUMAN**	**Q9NYB0**	**TERF2IP**	**0,0494** [a c]	0,02784	0,01318	0,01259	0,01054	4,64 - 44404	2,00e-07	110		9/21	32
**24**	**Creatine kinase B-type**	**KCRB_HUMAN**	**P12277**	**CKB**	**0,0011** [b c]	0,01997	0,02477	0,04969	0,01753	5,34 - 42902	1.3e-13	172		9/13	33
**25**	**Protein TFG**	**TFG_HUMAN**	**Q92734**	**TFG**	**0,0265** [a c]	0,02124	0,01006	0,00718	0,00554	4,94 - 43478	1.3e-07	112		8/12	28
**26**	**Adenylosuccinate lyase**	**PUR8_HUMAN**	**P30566**	**ADSL**	**0,0015** [c]	0,01279	0,02033	0,04793	0,03676	6,69 - 55595	6.5e-08	115		7/14	23
**27**	**ATP synthase subunit alpha, mitochondrial**	**ATPA_HUMAN**	**P25705**	**ATP5F1A**	**0,0094** [b]	0,02488	0,02887	0,04966	0,02451	9,16 - 59828	5.1e-17	206		12/15	30
**28**	**Ataxin-10**	**ATX10_HUMAN**	**Q9UBB4**	**ATXN10**	**0,0479** [d]	0,04331	0,04086	0,06492	0,11096	5,12 - 54196	2,00e-14	180		13/16	28
**29**	**Adenosylhomocysteinase**	**SAHH_HUMAN**	**P23526**	**AHCY**	**0,0031** [c]	0,02576	0,01966	0,05701	0,03000	5,92 - 48255	0.0017	120	71	8/30	17
	**Lupus La protein**	**LA_HUMAN**	**P05455**	**SSB**						6,68 - 46979	3.3e-05		88	10/30	28
**30**	**S-adenosylmethionine synthase isoform type-2**	**METK2_HUMAN**	**P31153**	**MAT2A**	**1,59e-05** [b c d]	0,01472	0,01426	0,06696	0,02954	6,02 - 43975	6.5e-07	213	105	11/42	39
	**Armadillo repeat-containing protein 6**	**ARMC6_HUMAN**	**Q6NXE6**	**ARMC6**						5,83 - 55191	4.1e-10		137	14/42	35

**31**	**Alpha-enolase**	**ENOA_HUMAN**	**P06733**	**ENO1**	**0,0026** [c]	0,09758	0,08111	0,04418	0,07295	7,01 - 47481	1.6e-26	301	19/19	55
**32**	**Rab GDP dissociation inhibitor beta**	**GDIB_HUMAN**	**P50395**	**GDI2**	**0,0314** [c]	0,03350	0,03152	0,06836	0,05679	6,11 - 51087	1.6e-14	181	16/34	48
**33**	**DAZ-associated protein 1**	**DAZP1_HUMAN**	**Q96EP5**	**DAZAP1**	**0,0263** [b]	0,06296	0,04536	0,09243	0,03824	8,73 - 43584	5.1e-10	136	11/15	29
**34**	**60 kDa heat shock protein, mitochondrial**	**CH60_HUMAN**	**P10809**	**HSPD1**	**0,0051** [c]	0,01616	0,02567	0,03702	0,02347	5,70 - 61187	5.1e-16	196	16/20	31
**35**					**0,0009** [b c]	0,01136	0,02219	0,04920	0,01412					
**36**	**Heterogeneous nuclear ribonucleoprotein D0**	**HNRPD_HUMAN**	**Q14103**	**HNRNPD**	**0,0005** [b d]	0,02787	0,02770	0,02316	0,09737	7,62 - 38581	0.00011	83	7/18	22
**37**	**Aspartate aminotransferase, cytoplasmic**	**AATC_HUMAN**	**P17174**	**GOT1**	**0,0463** [b]	0,06490	0,07200	0,04178	0,09153	6,52 - 46447	1.3e-28	322	21/27	64
**38**	**Isocitrate dehydrogenase [NADP] cytoplasmic**	**IDHC_HUMAN**	**O75874**	**IDH1**	**0,0192** [b d]	0,03516	0,03683	0,04080	0,01180	6,53 - 46915	8.1e-23	264	22/36	54
**39**	**Proliferation-associated protein 2G4**	**PA2G4_HUMAN**	**Q9UQ8 0**	**PA2G4**	**0,0329** [d]	0,01683	0,02139	0,01541	0,01005	6,13 - 44101	4.1e-09	127	13/22	38
**40**	**Aminoacylase-1**	**ACY1_HUMAN**	**Q03154**	**ACY1**	**0,0431** [d]	0,00834	0,01320	0,00641	0,00612	5,77 - 46084	3.8e-05	87	6/10	23
**41**	**Alpha-enolase**	**ENOA_HUMAN**	**P06733**	**ENO1**	**0,0094** [b]	0,05336	0,07763	0,02955	0,06128	7,01 - 47481	1.3e-21	252	20/25	54
**42**	**Glutamine synthetase**	**GLNA_HUMAN**	**P15104**	**GLUL**	**0,0419** [a c]	0,02193	0,04950	0,02237	0,02346	6,43 - 42665	5.1e-10	136	11/19	29
**43**	**Pyruvate dehydrogenase E1 component subunit alpha, somatic form, mitochondrial**	**ODPA_HUMAN**	**P08559**	**PDHA1**	**0,0004** [c]	0,02491	0,04435	0,05680	0,03361	8,35 - 43952	1.3e-16	202	17/26	36
**44**	**Creatine kinase U-type, mitochondrial**	**KCRU_HUMAN**	**P12532**	**CKMT1A; CKMT1B**	**0,0164** [a d]	0,13633	0,03997	0,13070	0,16247	8,60 - 47406	2.6e-17	209	17/22	43
**45**	**Short/branched chain specific acyl-CoA dehydrogenase, mitochondrial**	**ACDSB_HUMAN**	**P45954**	**ACADSB**	**0,0148** [b d]	0,01367	0,01103	0,00971	0,00442	6,53 - 47797	2.6e-13	169	12/19	41
**47**	**Fructose-bisphosphate aldolase A**	**ALDOA_HUMAN**	**P04075**	**ALDOA**	**0,0010** [a b d]	0,03869	0,26851	0,06377	0,01511	8,30 - 39851	1,00e-07	113	7/10	32

**48**	**Fructose-bisphosphate aldolase A**	**ALDOA_HUMAN**	**P04075**	**ALDOA**	**0,0254** [a b d]	0,04793	0,16569	0,07576	0,01263	8,30 - 39851	3.2e-12	158		10/12	42
**49**	**Heterogeneous nuclear ribonucleoproteins A2/B1**	**ROA2_HUMAN**	**P22626**	**HNRNPA2B1**	**0,0311** [b d]	0,11361	0,22613	0,07759	0,03614	8,97 - 37464	3.2e-19	228		15/18	51
**50**	**Biliverdin reductase A**	**BIEA_HUMAN**	**P53004**	**BLVRA**	**0,0414** [c d]	0,04219	0,04387	0,01355	0,01872	6,06 - 33692	1.3e-18	222		14/18	45
**51**	**Serine/threonine-protein phosphatase PP1-gamma catalytic subunit**	**PP1G_HUMAN**	**P36873**	**PPP1CC**	**0,0099** [a]	0,09840	0,04829	0,06895	0,04752	6,12 - 37701	1,00e-16	203		13/18	42
**52**	**Protein disulfide-isomerase A6**	**PDIA6_HUMAN**	**Q15084**	**PDIA6**	**0,0248** [c]	0,03694	0,02385	0,01378	0,02111	4,95 - 48490	2,00e-13	170		10/11	29
**53**	**Pyrroline-5-carboxylate reductase 2**	**P5CR2_HUMAN**	**Q96C36**	**PYCR2**	**0,0333** [d]	0,04926	0,02521	0,07091	0,12430	7.66 - 33958	1.3e-11	152		11/17	40
**56**	**Heterogeneous nuclear ribonucleoprotein A1**	**ROA1_HUMAN**	**P09651**	**HNRNPA1**	**0,0034** [c d]	0,11602	0,07724	0,05472	0,03355	9.17 - 38837	5.1e-23	266		17/22	48
**57**	**Voltage-dependent anion-selective channel protein 2**	**VDAC2_HUMAN**	**P45880**	**VDAC2**	**0,0023** [c d]	0,06128	0,09695	0,01207	0,01256	7,49 - 32060	4.1e-12	157		11/23	54
**58**	**3-mercaptopyruvate sulfurtransferase**	**THTM_HUMAN**	**P25325**	**MPST**	**0,0400** [b]	0,01466	0,01394	0,02520	0,01149	6,13 - 33443	1.3e-11	152		9/12	44
**59**	**Alpha-enolase N-term fragment**	**ENOA_HUMAN**	**P06733**	**ENO1**	**0,0075** [c d]	0,00974	0,00943	0,05038	0,08452	7.01 - 47481	4.1e-14	177		17/25	38
**60**	**Voltage-dependent anion-selective channel protein 1**	**VDAC1_HUMAN**	**P21796**	**VDAC1**	**0,0148** [a d]	0,07861	0,03306	0,08936	0,07571	8,62 - 30868	0.00021	80		5/9	29
**61**	**Electron transfer flavoprotein subunit alpha, mitochondrial**	**ETFA_HUMAN**	**P13804**	**ETFA**	**0,0143** [a c d]	0,02705	0,20008	0,01205	0,01169	8,62 - 35400	4.1e-10	175	137	13/36	42
	**Prohibitin-2**	**PHB2_HUMAN**	**Q99623**	**PHB2**						9,83 - 33276	0.0006		75	8/36	33
**62**	**Heterogeneous nuclear ribonucleoproteins A2/B1 *manca N-term***	**ROA2_HUMAN**	**P22626**	**HNRNPA2B1**	**0,0001** [a d]	0,03945	0,11503	0,06636	0,03412	8,97 - 37464	6.5e-24	275		19/23	49
**63**	**Proteasome activator complex subunit 3**	**PSME3_HUMAN**	**P61289**	**PSME3**	**0,0228** [d]	0,02822	0,04445	0,02087	0,01799	5,69 - 29602	0.0039	67		4/6	25

**64**	**tRNA (adenine(58)-N(1))-methyltransferase catalytic subunit TRMT61A**	**TRM61_HUMAN**	**Q96FX7**	**TRMT61A**	**0,0087** [a d]	0,02996	0,07735	0,02210	0,01782	6,89 - 31704	5.6e-05	86	6/14	34
**65**	**Sulfotransferase 1A1**	**ST1A1_HUMAN**	**P50225**	**SULT1A1**	**0,0455** [b d]	0,02448	0,02302	0,02450	0,00912	6,16 - 34257	6.5e-11	145	11/24	48
**66**					**0,0459** [a d]	0,00718	0,01639	0,00587	0,00586					
**67**					**0,0214** [c]	0,00991	0,01351	0,02262	0,01337					
**68**	**Fructose-bisphosphate aldolase A**	**ALDOA_HUMAN**	**P04075**	**ALDOA**	**0,0237** [a]	0,01049	0,02403	0,01379	0,01750	8,30 - 39851	0.0031	68	7/15	26
**69**					**0,0120** [a c d]	0,00154	0,01799	0,00373	0,00434					
**70**	**26S proteasome non-ATPase regulatory subunit 9**	**PSMD9_HUMAN**	**O00233**	**PSMD9**	**0,0039** [d]	0,01282	0,02268	0,00821	0,00454	6,46 - 24838	0.0085	64	5/7	21
**71**					**0,0062** [d]	0,00333	0,00663	0,00167	0,00154					
**72**	**Elongation factor 2** ***C-term fragment***	**EF2_HUMAN**	**P13639**	**EEF2**	**0,0013** [a d]	0,00201	0,01420	0,00217	0,00150	6,41 - 96246	2,00e-06	100	10/15	13
**73**					**0,0156** [a c d]	0,00918	0,02825	0,01878	0,01143					
**74**	**Prohibitin 1**	**PHB1_HUMAN**	**P35232**	**PHB1**	**0,0001** [b d]	0,11465	0,12876	0,09645	0,04515	5,57 - 29843	1.6e-26	301	16/17	76
**75**	**Elongation factor 2** ***C-term fragment***	**EF2_HUMAN**	**P13639**	**EEF2**	**0,0047** [c d]	0,01203	0,01796	0,00484	0,00372	6,41 - 96246	6.5e-07	105	8/10	13
**76**	**Phosphoglycerate mutase 1**	**PGAM1_HUMAN**	**P18669**	**PGAM1**	**0,0003** [a c]	0,02490	0,05818	0,06280	0,03412	6,67 - 28900	6.5e-15	185	10/12	61
**77**	**Heat shock 70 kDa protein 1A/ Heat shock 70 kDa protein 1B** ***N-term fragment***	**HS71A_HUMAN/ HS71B_HUMAN**	**P0DMV8/ P0DMV9**	**HSPA1A/ HSPA1B**	**0,0016** [c d]	0,02283	0,02209	0,16121	0,10711	5,48 - 70294	2,00e-14	180	12/13	19
**79**	**Heat shock 70 kDa protein 1A/ Heat shock 70 kDa protein 1B** ***N-term fragment***	**HS71A_HUMAN/ HS71B_HUMAN**	**P0DMV8/ P0DMV9**	**HSPA1A/ HSPA1B**	**0,0006** [b]	0,01411	0,01849	0,00889	0,02205	5,48 - 70294	2.4e-06	99	9/18	14
**80**	**Proteasome subunit alpha type-6**	**PSA6_HUMAN**	**P60900**	**PSMA6**	**0,0008** [a b d]	0,00569	0,02658	0,00935	0,00339	6,34 - 27838	5.1e-08	116	8/14	41
**81**	**Triosephosphate isomerase**	**TPIS_HUMAN**	**P60174**	**TPI1**	**0,0092** [a d]	0,00878	0,03111	0,00732	0,00791	6,45 - 26938	5.1e-09	126	9/21	35
**82**	**Peroxiredoxin-6**	**PRDX6_HUMAN**	**P30041**	**PRDX6**	**0,0149** [c]	0,03178	0,03977	0,07499	0,05670	6,00 - 25133	1.6e-05	91	6/13	30
**83**	**Kinetochore protein Spc25**	**SPC25_HUMAN**	**Q9HBM1**	**SPC25**	**0,0011** [a d]	0,02025	0,05840	0,01430	0,00739	7,71 - 26194	0.00016	81	6/11	29

**84**	**Serine/arginine-rich splicing factor 9**	**SRSF9_HUMAN**	**Q13242**	**SRSF9**	**0,0373** [c d]	0,01613	0,02406	0,00441	0,00555	8,74 - 25640	8.1e-11	144		10/12	40
**85**	**Protein-L-isoaspartate(D-aspartate) O-methyltransferase**	**PIMT_HUMAN**	**P22061**	**PCMT1**	**0,0224** [d]	0,02641	0,03759	0,01783	0,01572	6,70 - 24792	6.5e-10	135		9/13	38
**86**	**Proteasome subunit alpha type-2**	**PSA2_HUMAN**	**P25787**	**PSMA2**	**0,0384** [c d]	0,04539	0,05249	0,02262	0,01235	6,92 - 25996	2,00e-12	274	160	12/29	54
	**GTP-binding nuclear protein Ran**	**RAN_HUMAN**	**P62826**	**RAN**						7,01 - 24579	1.3e-09		132	10/29	43
**88**	**GTP-binding nuclear protein Ran**	**RAN_HUMAN**	**P62826**	**RAN**	**0,0071** [b d]	0,05823	0,07311	0,05383	0,02432	7,01 - 24579	1,00e-18	223		13/17	59
**89**	**Proteasome subunit beta type-3**	**PSB3_HUMAN**	**P49720**	**PSMB3**	**6,58e-07** [b]	0,04968	0,03248	0,09243	0,03795	6,14 - 23219	1.6e-12	161		10/11	40
**90**	**Glutathione S-transferase P**	**GSTP1_HUMAN**	**P09211**	**GSTP1**	**0,0007** [a b c d]	0,01180	0,03815	0,03841	0,01017	5,43 - 23569	4.1e-08	117		8/13	42
**91**	**Peroxiredoxin-1**	**PRDX1_HUMAN**	**Q06830**	**PRDX1**	**0,0042** [c d]	0,01970	0,03175	0,00805	0,00702	8,27 - 22324	1,00e-05	93		6/13	39
**92**	**Superoxide dismutase [Mn], mitochondrial**	**SODM_HUMAN**	**P04179**	**SOD2**	**0,0034** [a]	0,07830	0,18257	0,10836	0,09956	8,35 - 24906	1.3e-12	162		9/12	45
**93**	**Stromal cell-derived factor 2-like protein 1**	**SDF2L_HUMAN**	**Q9HCN8**	**SDF2L1**	**0,0157** [c d]	0,01390	0,02135	0,00662	0,00546	6,52 - 23812	3.4e-05	88		4/4	35
**94**	**Lactoylglutathione lyase**	**LGUL_HUMAN**	**Q04760**	**GLO1**	**0,0232** [b d]	0,15588	0,15287	0,08261	0,03963	5,12 - 20992	1,00e-13	173		12/20	66
**95**	**Transgelin-2**	**TAGL2_HUMAN**	**P37802**	**TAGLN2**	**0,0064** [a c]	0,08493	0,01194	0,00779	0,01132	8,41 - 22548	6.5e-23	265		19/26	88
**96**					**0,0001** [b c]	0,00361	0,00429	0,01952	0,00731						
**98**					**0,0158** [a c d]	0,00463	0,02241	0,00149	0,00274						
**99**					**0,0049** [a d]	0,00903	0,05328	0,00478	0,00794						
**100**	**Eukaryotic translation elongation factor 1 epsilon-1**	**MCA3_HUMAN**	**O43324**	**EEF1E1**	**0,0016** [a d]	0,18193	0,40562	0,19679	0,14310	8,54 - 19855	1.3e-11	152		9/15	44
**101**	**Nucleoside diphosphate kinase B**	**NDKB_HUMAN**	**P22392**	**NME2**	**0,0183** [c]	0,05314	0,07140	0,16440	0,10905	8,52 - 17401	5.1e-17	206		14/20	78
**102**					**0,0022** [a d]	0,00591	0,06278	0,00687	0,00522						
**103**	**Peptidyl-prolyl cis-trans isomerase A**	**PPIA_HUMAN**	**P62937**	**PPIA**	**0,0004** [a d]	0,09906	0,31140	0,07975	0,07720	7,68 - 18229	5.1e-13	166		10/13	47
**104**	**Peptidyl-prolyl cis-trans isomerase A**	**PPIA_HUMAN**	**P62937**	**PPIA**	**0,0301** [a d]	0,01502	0,03841	0,01040	0,00981	7,68 - 18229	8.9e-05	84		5/10	39

**105**	**Peptidyl-prolyl cis-trans isomerase A**	**PPIA_HUMAN**	**P62937**	**PPIA**	**0,0227** [a d]	0,00612	0,04664	0,00334	0,00333	7,68 - 18229	2,00e-05	90	5/8	43
**106**					**3,06e-07** [b d]	0,00855	0,01073	0,01212	0,04164					
**107**	**Mitochondrial fission 1 protein**	**FIS1_HUMAN**	**Q9Y3D6**	**FIS1**	**0,0028** [a c d]	0,01610	0,05859	0,03604	0,02866	8,84 - 16984	0.003	68	4/6	25
**108**	**Peptidyl-prolyl cis-trans isomerase-like 3**	**PPIL3_HUMAN**	**Q9H2H8**	**PPIL3**	**0,0487** [a d]	0,00523	0,01391	0,00279	0,00314	6,29 - 18371	4.1e-12	157	8/10	58
**109**	**Single-stranded DNA-binding protein, mitochondrial**	**SSBP_HUMAN**	**Q04837**	**SSBP1**	**0,0481** [c]	0,06595	0,06428	0,02589	0,05061	9,59 - 17249	3.2e-16	198	12/15	66
**110**	**Ubiquitin-conjugating enzyme E2 D3**	**UB2D3_HUMAN**	**P61077**	**UBE2D3**	**0,0119** [a d]	0,03955	0,01569	0,04789	0,06083	7,67 - 16904	6.5e-08	115	7/14	40
**111**	**Phosphatidylethanolamine-binding protein 1**	**PEBP1_HUMAN**	**P30086**	**PEBP1**	**0,0027** [b c d]	0,01924	0,03428	0,00464	0,00942	7,01 - 21158	8.1e-12	154	8/12	64
**112**					**0,0090** [a b c]	0,00836	0,02331	0,03542	0,01704					
**113**	**Single-stranded DNA-binding protein, mitochondrial**	**SSBP_HUMAN**	**Q04837**	**SSBP1**	**0,0076** [a b c]	0,06171	0,01697	0,01248	0,03157	9,59 - 17249	2.6e-16	199	12/18	70
**114**	**Profilin-1**	**PROF1_HUMAN**	**P07737**	**PFN1**	**0,0031** [a b c d]	0,20865	0,03377	0,05511	0,16513	8,44 - 15216	2.6e-12	159	9/11	63
**115**	**Small ribosomal subunit protein eS12**	**RS12_HUMAN**	**P25398**	**RPS12**	**0,0094** [c]	0,17441	0,18731	0,07701	0,14843	6,81 - 14905	1.1e-05	93	6/14	49
**116**	**NHP2-like protein 1**	**NH2L1_HUMAN**	**P55769**	**SNU13**	**0,0150** [a c]	0,01453	0,03978	0,04784	0,03883	8,72 - 14393	1.3e-09	132	7/11	51
**117**	**Thioredoxin**	**THIO_HUMAN**	**P10599**	**TXN**	**0,0022** [a c d]	0,13475	0,32672	0,02543	0,03226	4,82 - 12015	8.1e-09	124	9/17	72
**118**	**Eukaryotic translation initiation factor 1b**	**EIF1B_HUMAN**	**O60739**	**EIF1B**	**0,0140** [b c]	0,02188	0,01857	0,00643	0,01525	6,82 - 12930	2,00e-07	110	5/7	49
**119**					**0,0442** [a d]	0,03338	0,08600	0,01911	0,02788					
**120**					**0,0075** [d]	0,01819	0,02634	0,01420	0,00894					
**121**	**c-Myc-binding protein**	**MYCBP_HUMAN**	**Q99417**	**MYCBP**	**0,0004** [a d]	0,04679	0,10567	0,05127	0,04892	5,71 - 11959	5.1e-10	136	7/11	68
**122**	**Mitochondrial import inner membrane translocase subunit Tim9**	**TIM9_HUMAN**	**Q9Y5J7**	**TIMM9**	**0,0173** [a d]	0,00192	0,02024	0,00172	0,00279	6,71 - 10599	3.6e-05	88	4/5	61
**123**					**4,03e-05** [b d]	0,00989	0,01267	0,00587	0,03780					

**124**					**2,26e-06** [a b d]	0,00512	0,01152	0,00438	0,03597						
**125**	**Mitochondrial import inner membrane translocase subunit Tim10**	**TIM10_HUMAN**	**P62072**	**TIMM10**	**0,0109** [a d]	0,02272	0,09797	0,01622	0,01849	5,89 - 10554	2,00e-08	120		8/12	68
**126**	**Polyubiquitin-B [free Ubiquitin - monomer]**	**UBB_HUMAN**	**P0CG47**	**UBB**	**0,0001** [c]	0,35834	0,48285	0,85066	0,52840	6,86 - 25803	1.3e-06	102		6/7	23
**127**	**D-dopachrome decarboxylase**	**DOPD_HUMAN**	**P30046**	**DDT**	**0,0028** [c]	0,11587	0,06834	0,03631	0,03603	6,71 - 12818	3.2e-08	118		7/18	55
**128**	**Methanethiol oxidase**	**SBP1_HUMAN**	**Q13228**	**SELENBP1**	**0,0438** [c]	0,02611	0,01651	0,00951	0,01088	5,93 - 52928	2.6e-17	209		16/27	44
**129**	**Ornithine aminotransferase, mitochondrial**	**OAT_HUMAN**	**P04181**	**OAT**	**0,0216** [d]	0,10903	0,14499	0,07026	0,05823	6,57 - 48846	0.00087	287	74	9/42	28
	**Tubulin beta-4B chain**	**TBB4B_HUMAN**	**P68371**	**TUBB4B**						4,79 - 50255	1.3e-19		232	24/42	46
**130**	**Voltage-dependent anion-selective channel protein 1**	**VDAC1_HUMAN**	**P21796**	**VDAC1**	**0,0017** [a c d]	0,05546	0,14295	0,02499	0,01334	8,62 - 30868	1.3e-20	242		14/18	67
**131**	**Serine/threonine-protein phosphatase PP1-alpha catalytic subunit**	**PP1A_HUMAN**	**P62136**	**PPP1CA**	**1,97e-08** [b c d]	0,02680	0,02536	0,21455	0,06168	5,94 - 38229	2,00e-17	210		12/14	47

Table S2. Proteomic data and identifications of differential spots in Dsup-/+ HEK293T cells after oxidative stress

Table reports the spot numbers corresponding to that in Figure S1, protein name when the protein was identified by MALDI-ToF MS, UniProt Entry name, accession number (AC), and gene name, the ANOVA Test and the mean of the %V of the specific spot in Dsup- and Dsup+ cells. The right part of the table is dedicated to Mascot Search Results such as pI and MW, Expect and Score, Matched Peptides and sequence Coverage (%). In particular, only a differential abundance (Fold Change) ≥ 1.8 was considered for differential spots, therefore the mean of the %V of the spot has been evidenced in grey when higher.

<table>
<tr><th rowspan="2">Spot n°</th><th rowspan="2">Protein name</th><th rowspan="2">UniProt Name</th><th rowspan="2">AC</th><th rowspan="2">Gene Name</th><th colspan="3">Anova test</th><th colspan="6">Mascot Search Results</th></tr>
<tr><th>Anova (p)</th><th>Dsup- (H_2O_2)</th><th>Dsup+ (H_2O_2)</th><th>pI - MW</th><th>Expect</th><th colspan="2">Score</th><th>Matched peptides</th><th>Coverage(%)</th></tr>
<tr><td>1</td><td>Neurofilament light polypeptide</td><td>NFL_HUMAN</td><td>P07196</td><td>NEFL</td><td>1,61E-04</td><td><u>0,05368</u></td><td>0,01932</td><td>4,64 - 61536</td><td>6.5e-34</td><td colspan="2">375</td><td>32/40</td><td>52</td></tr>
<tr><td>2</td><td>Transaldolase</td><td>TALDO_HUMAN</td><td>P37837</td><td>TALDO1</td><td>8,82E-04</td><td><u>0,01836</u></td><td>0,00820</td><td>6,36 - 37688</td><td>5.1e-09</td><td colspan="2">126</td><td>10/18</td><td>30</td></tr>
<tr><td>3</td><td>Peroxiredoxin-6</td><td>PRDX6_HUMAN</td><td>P30041</td><td>PRDX6</td><td>9,82E-04</td><td><u>0,11622</u></td><td>0,05066</td><td>6,00 - 25133</td><td>8.1e-35</td><td colspan="2">384</td><td>24/34</td><td>79</td></tr>
<tr><td rowspan="3">4</td><td>Sorting nexin-6</td><td>SNX6_HUMAN</td><td>Q9UNH7</td><td>SNX6</td><td rowspan="3">0,0012323</td><td rowspan="3">0,01028</td><td rowspan="3"><u>0,03427</u></td><td>5,81 - 46905</td><td>2.4e-05</td><td rowspan="3">312</td><td>89</td><td>13/55</td><td>34</td></tr>
<tr><td>Pyruvate kinase PKM</td><td>KPYM_HUMAN</td><td>P14618</td><td>PKM</td><td>7,96 - 58470</td><td>6.5e-08</td><td>115</td><td>15/55</td><td>32</td></tr>
<tr><td>Pachytene checkpoint protein 2 homolog</td><td>PCH2_HUMAN</td><td>Q15645</td><td>TRIP13</td><td>5,73 - 48863</td><td>1.6e-11</td><td>151</td><td>16/55</td><td>43</td></tr>
<tr><td>5</td><td>Acetyl-CoA acetyltransferase, mitochondrial</td><td>THIL_HUMAN</td><td>P24752</td><td>ACAT1</td><td>0,0014789</td><td><u>0,10677</u></td><td>0,04467</td><td>8,98 - 45456</td><td>2,00E-17</td><td colspan="2">210</td><td>16/23</td><td>48</td></tr>
<tr><td>6</td><td>NADH dehydrogenase [ubiquinone] 1 alpha subcomplex subunit 3</td><td>NDUA3_HUMAN</td><td>O95167</td><td>NDUFA3</td><td>0,0028296</td><td><u>0,02453</u></td><td>0,00867</td><td>7,28 - 9273</td><td>0.024</td><td colspan="2">59</td><td>3/7</td><td>54</td></tr>
<tr><td>7</td><td>7-methylguanosine phosphate-specific 5'-nucleotidase</td><td>5NT3B_HUMAN</td><td>Q969T7</td><td>NT5C3B</td><td>0,0035328</td><td><u>0,01635</u></td><td>0,00230</td><td>5,94 - 34653</td><td>0.00042</td><td colspan="2">77</td><td>6/14</td><td>28</td></tr>
<tr><td>8</td><td>V-type proton ATPase subunit F</td><td>VATF_HUMAN</td><td>Q16864</td><td>ATP6V1F</td><td>0,003839</td><td>0,00766</td><td><u>0,03090</u></td><td>5,29 - 13362</td><td>2,00E-16</td><td colspan="2">200</td><td>11/16</td><td>84</td></tr>
<tr><td>9</td><td>Enoyl-CoA delta isomerase 1, mitochondrial</td><td>ECI1_HUMAN</td><td>P42126</td><td>ECI1</td><td>0,0044011</td><td><u>0,03582</u></td><td>0,01096</td><td>8,80 - 33080</td><td>5.1e-13</td><td colspan="2">166</td><td>9/10</td><td>39</td></tr>
</table>

**10**	**Thioredoxin**	**THIO_HUMAN**	**P10599**	**TXN**	0,0056704	0,04129	**0,10965**	4,82 - 12015	3.2e-08	118	9/23	118
**11**	**Pyruvate kinase PKM**	**KPYM_HUMAN**	**P14618**	**PKM**	0,005703	**0,04978**	0,02457	7,96 - 58470	2,00E-29	330	26/35	46
**12**	**T-complex protein 1 subunit beta**	**TCPB_HUMAN**	**P78371**	**CCT2**	0,0057753	**0,03193**	0,01488	6,01 - 57794	2.6e-46	499	39/50	78
**13**	**Heat shock cognate 71 kDa protein**	**HSP7C_HUMAN**	**P11142**	**HSPA8**	0,0063351	**0,02568**	0,00750	5,37 - 71082	3.2e-13	168	12/16	29
**14**	**Ferritin light chain**	**FRIL_HUMAN**	**P02792**	**FTL**	0,0068109	0,00839	**0,01707**	5,51 - 20064	8.1e-11	144	8/11	54
**15**	**G-rich sequence factor 1**	**GRSF1_HUMAN**	**Q12849**	**GRSF1**	0,0072079	**0,06831**	0,02587	5,83 - 53606	2,00E-19	230	20/29	49
**16**	**Heterogeneous nuclear ribonucleoproteins A2/B1**	**ROA2_HUMAN**	**P22626**	**HNRNPA2B1**	0,0076834	0,00271	**0,01340**	8,97 - 37464	1.3e-06	102	7/16	31
**17**	**Cytochrome c oxidase subunit 6B1**	**CX6B1_HUMAN**	**P14854**	**COX6B1**	0,0078464	0,00418	**0,02072**	6,54 - 10414	4.8e-05	86	5/11	65
**18**	**Thioredoxin-dependent peroxide reductase, mitochondrial**	**PRDX3_HUMAN**	**P30048**	**PRDX3**	0,0079654	**0,09085**	0,03826	7,67 - 28017	4.1e-14	177	13/26	62
**19**	**Heterogeneous nuclear ribonucleoprotein H**	**HNRH1_HUMAN**	**P31943**	**HNRNPH1**	0,0086397	0,02570	**0,06632**	5,89 - 49484	2,00E-16	200	13/17	49
**20**	**Ubiquitin-conjugating enzyme E2 C**	**UBE2C_HUMAN**	**O00762**	**UBE2C**	0,0087522	**0,01719**	0,00833	6,82 - 19754	1.3e-20	242	13/15	79
**21**					0,0112534	0,00984	**0,02101**					
**22**	**Cytoplasmic aconitate hydratase**	**ACOHC_HUMAN**	**P21399**	**ACO1**	0,0126418	**0,01262**	0,00350	6,23 - 98850	2,00E-21	250	19/23	34
**23**	**5'(3')-deoxyribonucleotidase, cytosolic type**	**NT5C_HUMAN**	**Q8TCD5**	**NT5C**	0,0138241	**0,01898**	0,00709	6,18 - 23596	2,00E-11	150	10/16	60
**24**	**Guanosine-3',5'-bis(diphosphate) 3'-pyrophosphohydrolase MESH1**	**MESH1_HUMAN**	**Q8N4P3**	**HDDC3**	0,0154718	**0,01533**	0,00692	6,24 - 20374	6.5e-08	115	6/8	39

**25**	**Sorting and assembly machinery component 50 homolog**	**SAM50_HUMAN**	**Q9Y512**	**SAMM50**	0,0246064	**0,01971**	0,00805	6,44 - 52342	3.2e-12	158		12/20	32
**26**	**60 kDa heat shock protein, mitochondrial**	**CH60_HUMAN**	**P10809**	**HSPD1**	0,0252011	**0,03304**	0,01591	5,70 - 61187	4.1e-15	270	187	19/39	46
	**Heat shock 70 kDa protein 1A**	**HS71A_HUMAN**	**P0DMV8**	**HSPA1A**				5,48 - 70294	0.0023		70	9/39	22
**27**	**Heat shock 70 kDa protein 4L**	**HS74L_HUMAN**	**O95757**	**HSPA4L**	0,0277754	**0,01147**	0,00215	5,63 - 95479	0.0021	70		8/18	13
**28**	**Proteasome subunit alpha type-4**	**PSA4_HUMAN**	**P25789**	**PSMA4**	0,0311138	**0,04356**	0,00962	7,57 - 29750	1,00E-11	266	153	11/24	61
	**Adenylate kinase 2, mitochondrial**	**KAD2_HUMAN**	**P54819**	**AK2**				7,67 - 26689	3.2e-09		128	10/24	55
**29**	**Heat shock 70 kDa protein 1A/ Heat shock 70 kDa protein 1B *N-term fragment***	**HS71A_HUMAN/ HS71B_HUMAN**	**P0DMV8 / P0DMV9**	**HSPA1A/ HSPA1B**	0,0318424	0,00525	**0,01129**	5,48 - 70294	4.1e-09	127		10/16	22
**30**	**Serine/arginine-rich splicing factor 1**	**SRSF1_HUMAN**	**Q07955**	**SRSF1**	0,0321045	**0,07631**	0,02610	10,37 - 27842	5.1e-16	196		12/15	43
**31**	**Mannose-6-phosphate isomerase**	**MPI_HUMAN**	**P34949**	**MPI**	0,0350633	0,01209	**0,03916**	5,62 - 47196	2,00E-05	90		8/22	23
**32**	**Protein S100-A10**	**S10AA_HUMAN**	**P60903**	**S100A10**	2,55E-04	**0,04175**	0,02210	6,82 - 11310	0.0004	77		5/11	51
**33**	**Polyubiquitin-B [free Ubiquitin]**	**UBB_HUMAN**	**P0CG47**	**UBB**	0,0012832	**0,34286**	0,17861	6,86 - 25803	3.2e-09	128		11/24	28
**34**	**Polyubiquitin-B [free Ubiquitin]**	**UBB_HUMAN**	**P0CG47**	**UBB**	0,0015926	**0,16187**	0,08252	6,86 - 25803	1,00E-11	153		10/15	28
**35**	**Proteasome subunit alpha type-1**	**PSA1_HUMAN**	**P25786**	**PSMA1**	0,0017703	**0,02214**	0,00546	6,15 - 29822	2,00E-09	130		9/13	36
**36**	**Heat shock 70 kDa protein 1A/ Heat shock 70 kDa protein 1B**	**HS71A_HUMAN/ HS71B_HUMAN**	**P0DMV8 / P0DMV9**	**HSPA1A/ HSPA1B**	0,0020901	**0,07523**	0,02919	5,48 - 70294	4.1e-34	377		31/46	57
**37**	**Protein disulfide-isomerase A6**	**PDIA6_HUMAN**	**Q15084**	**PDIA6**	0,0026947	0,00196	**0,00702**	4,95 - 48490	3.2e-08	118		8/13	29

**38**	**Heat shock 70 kDa protein 1A/ Heat shock 70 kDa protein 1B**	**HS71A_HUMAN/ HS71B_HUMAN**	**P0DMV8 / P0DMV9**	**HSPA1A/ HSPA1B**	0,0028797	**0,01327**	0,00610	5,48 - 70294	3.2e-15	188		16/30	36
**39**	**Peroxiredoxin-1**	**PRDX1_HUMAN**	**Q06830**	**PRDX1**	0,0042314	0,01688	**0,06636**	8,27 - 22324	8.1e-23	264		15/22	74
**40**	**Alpha-enolase**	**ENOA_HUMAN**	**P06733**	**ENO1**	0,0042768	**0,02792**	0,01144	7,01 - 47481	2,00E-21	250		20/27	44
**41**	**Heterogeneous nuclear ribonucleoprotein A3**	**ROA3_HUMAN**	**P51991**	**HNRNPA3**	0,0047061	0,00087	**0,00651**	9,10 - 39799	5.1e-10	136		11/25	35
**42**	**Dual specificity mitogen-activated protein kinase kinase 2**	**MP2K2_HUMAN**	**P36507**	**MAP2K2**	0,0052203	**0,07181**	0,03817	6,12 - 44681	2,00E-13	170		14/28	33
**43**	**Nucleophosmin**	**NPM_HUMAN**	**P06748**	**NPM1**	0,0071538	0,03121	**0,07545**	4,64 - 32726	3.3e-05	88		8/15	27
**44**	**Phosphoglycerate mutase 1**	**PGAM1_HUMAN**	**P18669**	**PGAM1**	0,0077401	0,00265	**0,00654**	6,67 - 28900	8.1e-11	234	144	10/21	51
	**Myeloid leukemia factor 2**	**MLF2_HUMAN**	**Q15773**	**MLF2**				6,40 - 28186	4.1e-07		107	9/21	36
**45**	**Exocyst complex component 7**	**EXOC7_HUMAN**	**Q9UPT5**	**EXOC7**	0,0102289	0,00170	**0,00387**	6,33 - 83729	1.6e-11	151		13/18	22
**46**	**Ezrin**	**EZRI_HUMAN**	**P15311**	**EZR**	0,0108427	**0,00919**	0,00390	5,94 - 69484	5.1e-11	146		13/17	24
**47**	**Ubiquitin carboxyl-terminal hydrolase isozyme L3**	**UCHL3_HUMAN**	**P15374**	**UCHL3**	0,0132945	0,01887	**0,12458**	4,84 - 26337	6.5e-18	215		13/17	69
**48**	**Proteasome activator complex subunit 3**	**PSME3_HUMAN**	**P61289**	**PSME3**	0,0138022	0,00910	**0,01813**	5,69 - 29602	0.0056	66		5/13	28
**49**	**60S acidic ribosomal protein P0**	**RLA0_HUMAN**	**P05388**	**RPLP0**	0,0139973	0,06985	**0,12757**	5,71 - 34423	6.5e-12	277	155	12/26	47
	**Tubulin beta chain**	**TBB5_HUMAN**	**P07437**	**TUBB**				4,78 - 50095	1,00E-09		133	11/26	40
**50**	**3-mercaptopyruvate sulfurtransferase**	**THTM_HUMAN**	**P25325**	**MPST**	0,0148027	**0,01982**	0,01035	6,13 - 33443	1,00E-18	278	223	17/28	67
	**Proteasome subunit alpha type-1**	**PSA1_HUMAN**	**P25786**	**PSMA1**				6,15 - 29822	0.00072		74	7/28	36

**51**	**Peptidyl-prolyl cis-trans isomerase FKBP4**	**FKBP4_HUMAN**	**Q02790**	**FKBP4**	0,0169767	0,00332	**0,01025**	5,35 - 52057	4.1e-08	117		10/22	28
**52**	**Proteasome subunit alpha type-5**	**PSA5_HUMAN**	**P28066**	**PSMA5**	0,0177649	**0,09844**	0,02832	4,74 - 26565	1.6e-14	196	181	14/34	65
	**Tubulin beta chain *N-term fragment***	**TBB5_HUMAN**	**P07437**	**TUBB**				4,78 - 50095	0.01		63	7/34	26
**53**	**Proteasome subunit alpha type-1**	**PSA1_HUMAN**	**P25786**	**PSMA1**	0,0226659	0,01185	**0,04352**	6,15 - 29822	8.1e-08	114		8/15	39
**54**	**Mitogen-activated protein kinase 1**	**MK01_HUMAN**	**P28482**	**MAPK1**	0,0236102	**0,04993**	0,02568	6,50 - 41762	1,00E-07	113		8/13	23
**55**	**Pyruvate kinase PKM**	**KPYM_HUMAN**	**P14618**	**PKM**	0,0256442	0,04724	**0,08904**	7,96 - 58470	1,00E-19	324	233	22/37	41
	**T-complex protein 1 subunit theta**	**TCPQ_HUMAN**	**P50990**	**CCT8**				5,42 - 60153	1.8e-05		91	11/37	21
**56**	**S-adenosylmethionine synthase isoform type-2**	**METK2_HUMAN**	**P31153**	**MAT2A**	0,0293622	0,01137	**0,02231**	6,02 - 43975	0.0083	124	64	6/20	21
	**Heterogeneous nuclear ribonucleoprotein H**	**HNRH1_HUMAN**	**P31943**	**HNRNPH1**				5,89 - 49484	1,00E-05		93	8/20	31
**57**	**Proteasome subunit beta type-3**	**PSB3_HUMAN**	**P49720**	**PSMB3**	0,0335696	**0,05270**	0,02708	6,14 - 23219	4.1e-11	147		11/14	40
**58**	**Ataxin-10**	**ATX10_HUMAN**	**Q9UBB4**	**ATXN10**	0,041865	**0,02396**	0,01049	5,12 - 54196	2,00E-14	180		13/16	28
**59**	**D-dopachrome decarboxylase**	**DOPD_HUMAN**	**P30046**	**DDT**	0,0433558	**0,04428**	0,02407	6,71 - 12818	4.1e-16	197		11/22	57
**60**					0,0490693	0,00307	**0,00584**						
