# Supplementary material for: Investigating the Cytoprotective Mechanisms of the Tardigrade Damage Suppressor (Dsup) Protein in Human Cells Under Hypoxic Stress
Source: Int J Mol Sci. 2025 Oct 28;26(21):10452. doi: 10.3390/ijms262110452 (PMC12607402; doi:10.3390/ijms262110452)
Supplement: Supplementary file 1 [file ijms-26-10452-s001.zip › Supplementary figures hypoxia final.pptx]

## Slide 1
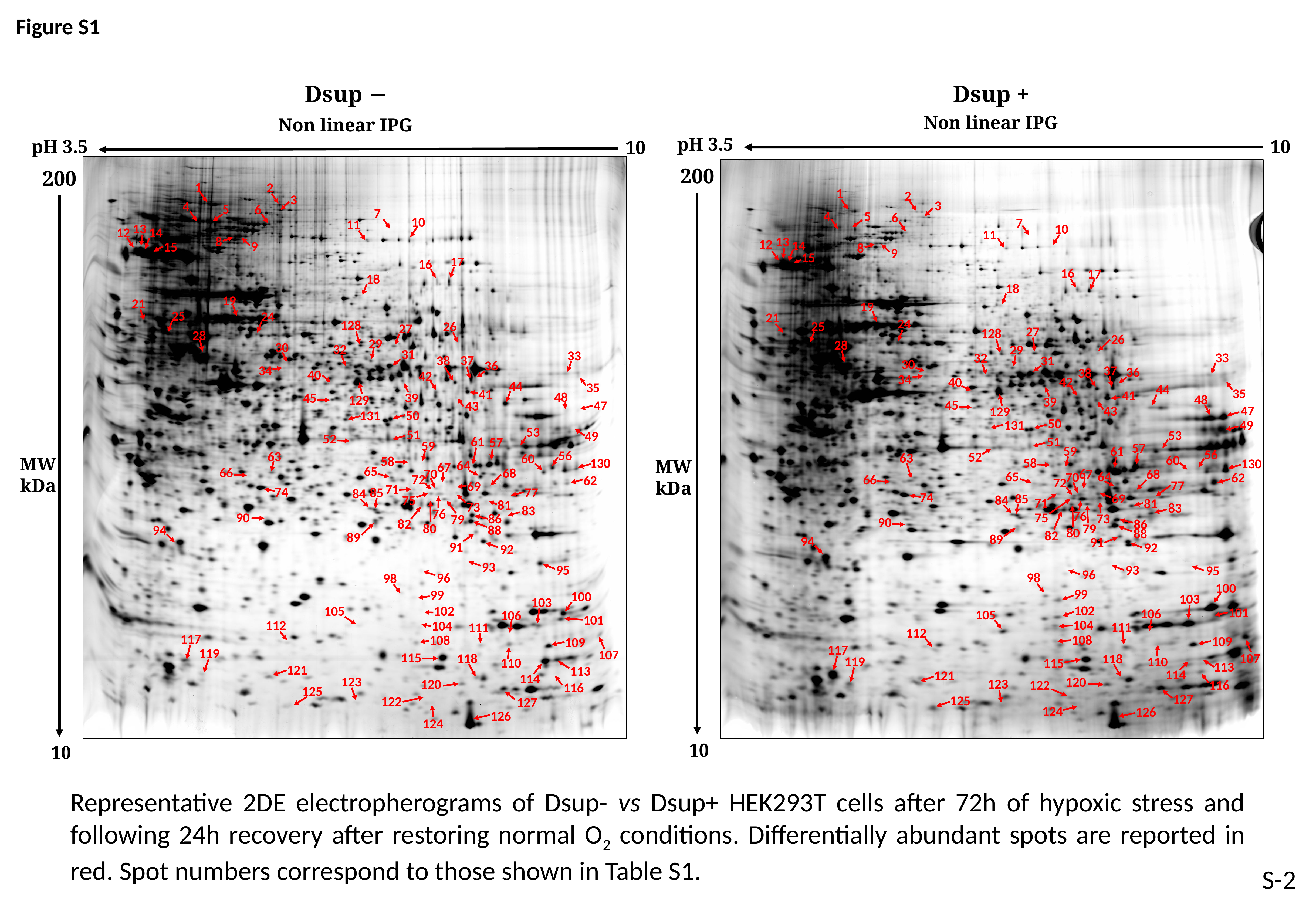

Figure S1
Dsup −
Dsup +
Non linear IPG
pH 3.5
10
Non linear IPG
pH 3.5
10
1
2
3
4
5
6
7
10
11
13
12
14
8
9
15
17
16
18
19
21
25
24
128
26
27
28
29
30
32
31
33
37
38
36
34
40
42
44
35
41
48
39
45
129
47
43
50
131
53
51
49
52
61
57
59
56
63
60
58
130
64
67
65
66
68
70
72
62
69
71
74
85
77
84
75
81
73
83
76
90
86
79
82
80
94
88
89
91
92
93
95
96
98
99
100
103
105
102
106
101
112
104
111
117
108
109
119
107
115
118
110
121
113
114
123
120
116
125
122
127
126
124
1
2
3
4
5
6
7
10
11
13
12
14
8
9
15
16
17
18
19
21
24
25
27
128
26
28
29
33
32
31
30
37
36
38
34
42
40
44
35
41
48
39
45
47
43
129
50
49
131
53
51
57
59
61
56
52
63
60
58
130
67
68
64
65
70
62
66
72
77
74
69
85
84
71
81
83
76
75
73
90
86
79
80
88
82
89
94
91
92
93
95
96
98
100
99
103
102
101
106
105
104
111
112
108
109
117
107
118
119
110
115
113
114
121
120
123
122
116
127
125
124
126
200
10
200
10
MW
kDa
MW
kDa
Representative 2DE electropherograms of Dsup- vs Dsup+ HEK293T cells after 72h of hypoxic stress and following 24h recovery after restoring normal O2 conditions. Differentially abundant spots are reported in red. Spot numbers correspond to those shown in Table S1.
S-2

## Slide 2
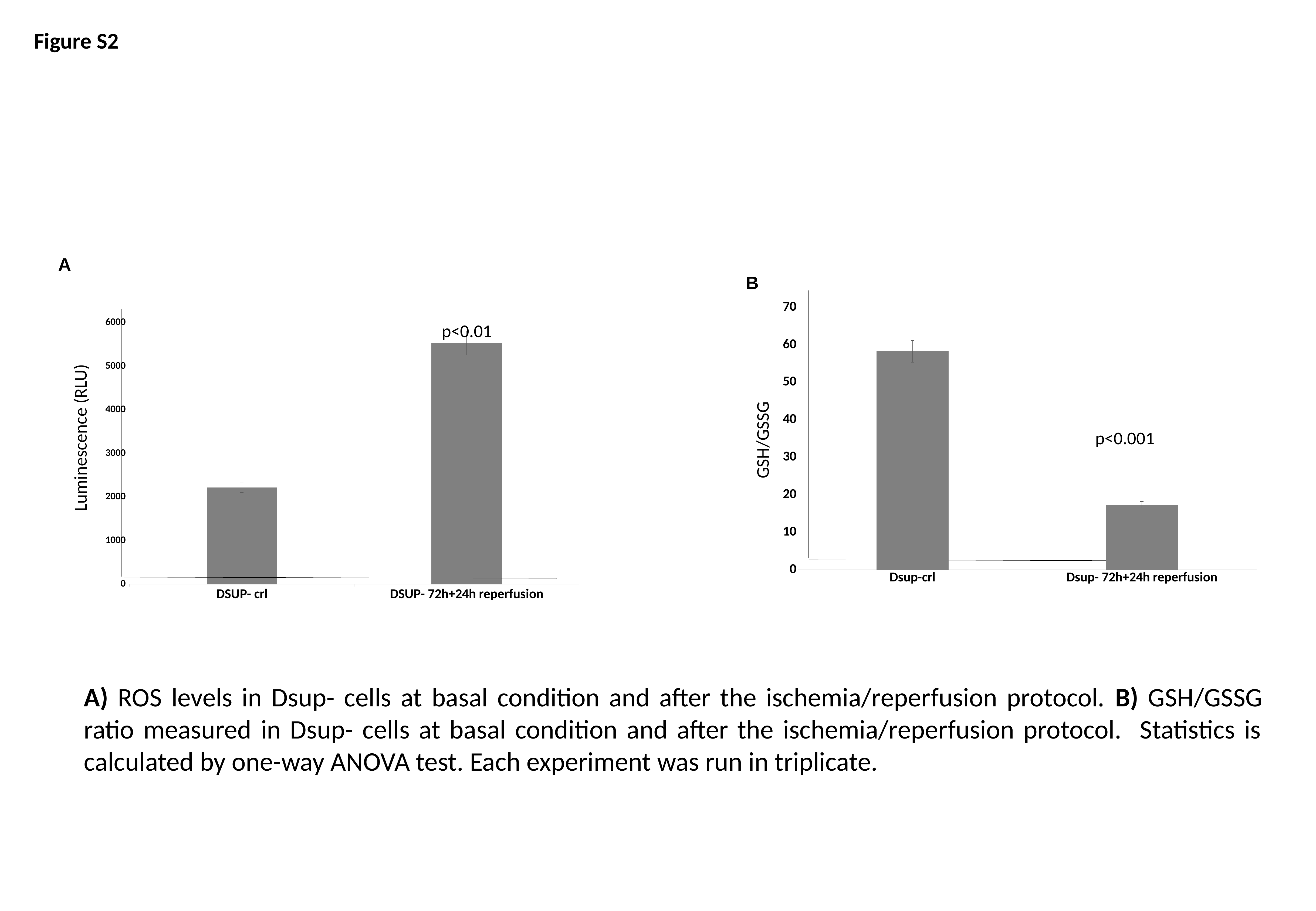

Figure S2
A
### Chart
| Category | |
|---|---|
| DSUP- crl | 2217.0 |
| DSUP- 72h+24h reperfusion | 5535.0 |p<0.01
Luminescence (RLU)
B
### Chart
| Category | |
|---|---|
| Dsup-crl | 58.26 |
| Dsup- 72h+24h reperfusion | 17.32 |
### Chart
| Category |
|---|GSH/GSSG
p<0.001
A) ROS levels in Dsup- cells at basal condition and after the ischemia/reperfusion protocol. B) GSH/GSSG ratio measured in Dsup- cells at basal condition and after the ischemia/reperfusion protocol. Statistics is calculated by one-way ANOVA test. Each experiment was run in triplicate.

## Slide 3
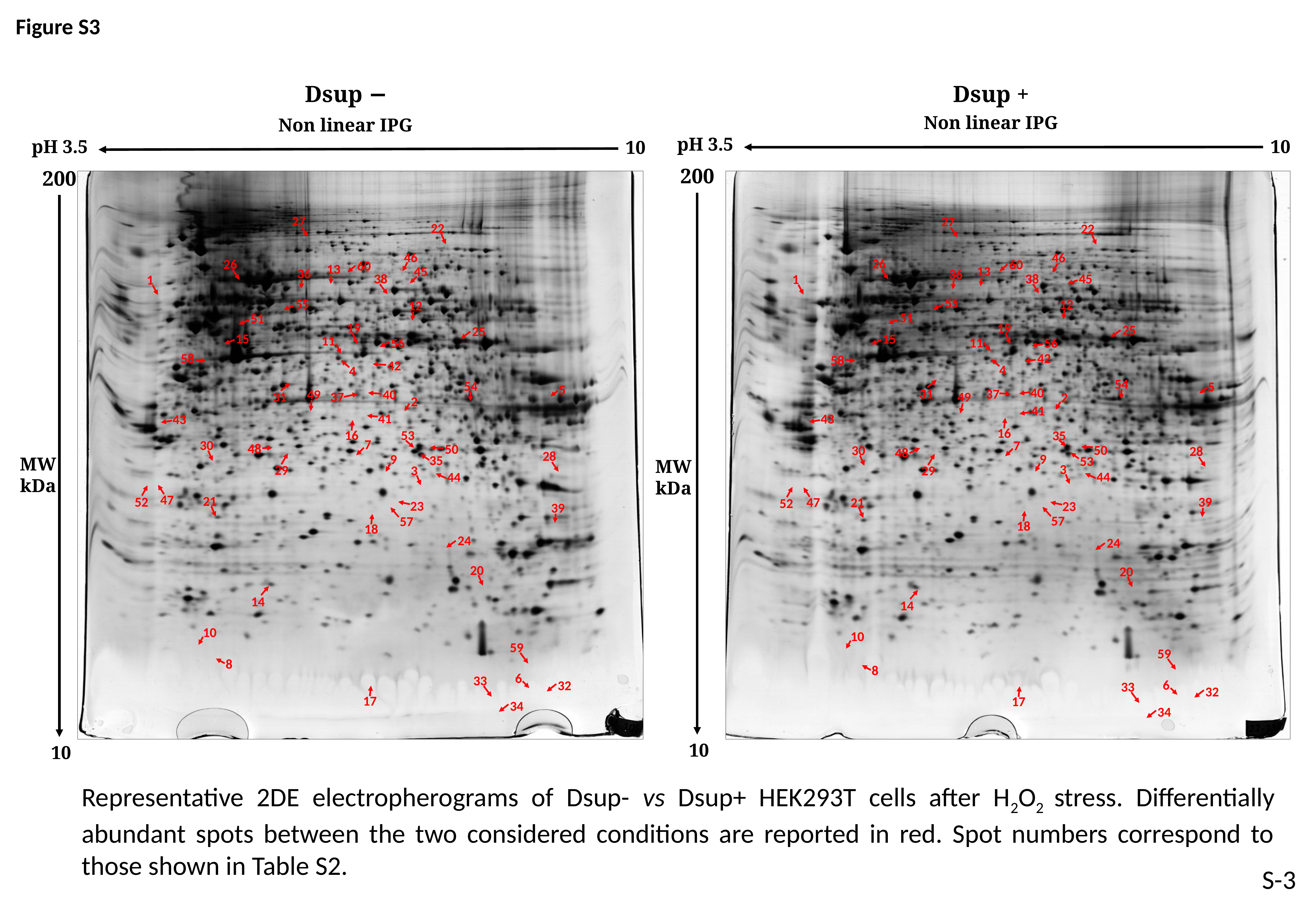

Figure S3
Dsup −
Dsup +
Non linear IPG
pH 3.5
10
Non linear IPG
pH 3.5
10
200
10
200
10
27
27
22
22
46
46
26
26
60
60
13
13
45
36
36
38
38
45
1
1
55
55
12
12
51
51
19
19
25
25
15
15
11
11
56
56
58
42
58
42
4
4
54
54
5
5
40
31
37
40
49
37
49
31
2
2
41
41
43
43
16
16
53
35
7
30
7
48
50
50
30
28
48
28
9
9
35
53
MW
kDa
MW
kDa
3
29
3
29
44
44
47
21
52
39
47
21
52
23
23
39
57
57
18
18
24
24
20
20
14
14
10
10
59
59
8
8
6
33
6
32
33
32
17
17
34
34
Representative 2DE electropherograms of Dsup- vs Dsup+ HEK293T cells after H2O2 stress. Differentially abundant spots between the two considered conditions are reported in red. Spot numbers correspond to those shown in Table S2.
S-3
